# Construction of anti-codon table of the plant kingdom and evolution of tRNA selenocysteine (tRNA^Sec^)

**DOI:** 10.1186/s12864-020-07216-3

**Published:** 2020-11-19

**Authors:** Tapan Kumar Mohanta, Awdhesh Kumar Mishra, Abeer Hashem, Elsayed Fathi Abd_Allah, Abdul Latif Khan, Ahmed Al-Harrasi

**Affiliations:** 1 Natural and Medical Sciences Research Center, University of Nizwa, 616 Nizwa, Oman; 2 Department of Biotechnology, Yeungnam University, 38541 Gyeongsan, South Korea; 3 Botany and Microbiology Department, College of Science, King Saud University, P.O. Box. 2460, Riyadh, 11451 Saudi Arabia; 4 Mycology and Plant Disease Survey Department, Plant Pathology Research Institute, ARC, Giza, 12511 Egypt; 5 Plant Production Department, College of Food and Agricultural Sciences, King Saud University, P.O. Box. 2460, Riyadh, 11451 Saudi Arabia

**Keywords:** tRNA, Evolution, Anti-codon, tRNA^Sec^, Protein translation, Wobble

## Abstract

**Background:**

The tRNAs act as a bridge between the coding mRNA and incoming amino acids during protein translation. The anti-codon of tRNA recognizes the codon of the mRNA and deliver the amino acid into the protein translation chain. However, we did not know about the exact abundance of anti-codons in the genome and whether the frequency of abundance remains same across the plant lineage or not.

**Results:**

Therefore, we analysed the tRNAnome of 128 plant species and reported an anti-codon table of the plant kingdom. We found that CAU anti-codon of tRNA^Met^ has highest (5.039%) whereas GCG anti-codon of tRNA^Arg^ has lowest (0.004%) abundance. However, when we compared the anti-codon frequencies according to the tRNA isotypes, we found tRNA^Leu^ (7.808%) has highest abundance followed by tRNA^Ser^ (7.668%) and tRNA^Gly^ (7.523%). Similarly, suppressor tRNA (0.036%) has lowest abundance followed by tRNA^Sec^ (0.066%) and tRNA^His^ (2.109). The genome of *Ipomoea nil, Papaver somniferum*, and *Zea mays* encoded the highest number of anti-codons (isoacceptor) at 59 each whereas the genome of *Ostreococcus tauri* was found to encode only 18 isoacceptors. The *tRNA*^*Sec*^ genes undergone losses more frequently than duplication and we found that *tRNA*^*Sec*^ showed anti-codon switch during the course of evolution.

**Conclusion:**

The anti-codon table of the plant tRNA will enable us to understand the synonymous codon usage of the plant kingdom and can be very helpful to understand which codon is preferred over other during the translation.

**Supplementary Information:**

The online version contains supplementary material available at 10.1186/s12864-020-07216-3.

## Background

The proteins present in cells are the product of the blueprint prescribed by the genes [1–3]. Collectively, all of the genes (including coding and non-coding) presents in a cell represent the genome of an organism [4, 5]. The construction of a protein from a gene is a complex procedure and requires the involvement of transfer RNA (tRNA), messenger RNA (mRNA), ribosomes, amino acids, and other molecules [6–9]. This process is commonly known as translation which is a fundamental parameter of living cells [6–9]. The functional apparatus involved in gene translation is highly conserved across the tree of life [10]. mRNA conveys the blueprint information as triplet codons composed of nucleotides and tRNA are able to perceive the cognate codons [11, 12]. Although mRNA and ribosomes represent the two major parts of the machinery responsible for translation, transfer RNAs (tRNAs) are the fundamental units of this translation machinery [13–15]. The anti-codon of a tRNA links to the codon of the mRNA and supplies the corresponding amino acid into the protein translation chain [3, 8, 15, 16]. Two or more different tRNAs can bind an amino acid and transfer it to the ribosome [17–20]. There are 22 different amino acids encoded by 63 codons (including UGA and UAG codons for selenocysteine and pyrrolysine, respectively) as several of the amino acids are encoded by more than one codon and hence its corresponding anti-codon [21–25]. Therefore, it is possible to encode more than one tRNA molecule with different anti-codons to transfer a particular amino acid [21, 26–28]. Although codon selection for a corresponding anti-codon is the primary unit of the translation machinery, mutational bias, selection, drift, and codon usage bias also shape the prescribed translation [29–32]. Although there are critical steps for the efficient and proper functioning of the translation machinery, other synonymous codons can also serve as an alternative choice [32–34]. The differential use of codons also reflects their natural demand in the protein translation machinery [35, 36]. tRNAs are classified into various gene families based on their isoacceptor anti-codons [17, 19, 20]. The available tRNA pool is maintained at a level that can accommodate the transcript levels present in a cell, thus ensuring efficient and accurate translation. Highly-expressed genes, however, exhibit codon usage bias that reflects the copy number of the corresponding tRNA [37–39]. Translational selection acts to maintain the balance between codon usage and tRNA availability [40–42]. There is always selection pressure, however, to increase the production of the codons used in highly-expressed genes [32, 43, 44].

Over the course of evolution, the earth has undergone enormous changes and the plant kingdom has been subjected to numerous stresses [45–47]. All living organisms had to adapt to a changing environment, which resulted in the increased importance of some protein-coding genes while others became less important [48–50]. Accordingly, there was a need to alter the relative number and type of available tRNAs to fulfil the translational requirements of the new and/or modified protein-coding genes [51, 52]. Changes in the relative number and type of tRNA molecules are also associated with a change in the number and type of anti-codons [53, 54]. The role of selection pressure brought about by translational demand and its role in maintaining tRNA pools has not been adequately addressed. Furthermore, the selection pressure that determines the maintenance of low copy tRNA families and anti-codons also remains unclear. Whether translational selection pressure favours optimal codons in particular cases and keeps other codons as non-optimal, and hence in low supply, is unknown. It is also unknown if the amino acid requirements of proteins impact the need to provide specific tRNAs having the required anti-codons, as well as the genes that encode those tRNAs. In the present study, an attempt was made to determine the frequency of anti-codons in the tRNAnome of the Plant Kingdom to better understand the presence of codons and anti-codon frequency. Our objective was to provide information on the link between the presence of codons and their corresponding anti-codons, tRNAs, and the number of amino acids utilized in plant proteomes. Therefore, we analysed the frequency of anti-codons in the tRNA of plant genomes and constructed an anti-codon table of the Plant Kingdom.

## Material and methods

### Sequence retrieval

The annotated RNA sequence files of all 128 plant species were downloaded from the National Center for Biotechnology Information (NCBI) using the Ensemble genome browser. The downloaded sequence files were scanned for the presence of tRNAs using tRNAscan-SE software on a Linux-based platform. The resulting tRNAscan files were used for further analysis. After the completion of the scanning of individual files, all files were merged to obtain a complete plant tRNAnome file. The frequency of each individual anti-codon was obtained from the tRNAnome file and presented as a number and percentage (%). In the course of the analysis, several tRNA^Sec^ were identified in different plant genomes and were kept separately for further study.

### Sequence alignment

Multiple sequence alignment of tRNA^Sec^ genes was conducted using multalin software with default parameters. To construct the phylogenetic tree, a multiple sequence alignment of tRNAs and tRNA^Sec^ were conducted using the MUSCLE program in MEGA7 software [55, 56]. The resulting alignment was saved in a MEGA file format. The alignment file was subsequently used to construct a phylogenetic tree using MEGA7 software. Prior to the construction of the phylogenetic tree, a model selection was carried out using the following statistical parameters; statistical method, maximum likelihood substitution type, nucleotides, gaps/missing data treatment, complete deletion. Based on the lowest BIC score, a phylogenetic tree of tRNAs and tRNA^Sec^ was constructed. The statistical parameters used to construct the phylogenetic tree were: statistical method (maximum likelihood), test of phylogeny (bootstrap method), no. of bootstrap replicates (1000), substitution type (nucleotides), model/method (Kimura-2-parameter model), rates among sites (gamma distributed), no. of discrete gamma parameters (5), gaps/missing data treatment (partial deletion), site coverage cut-off (95%), ML Heuristic method (nearest-neighbour-interchange), and branch swap filter (very strong). A separate phylogenetic tree was constructed using all of the tRNA^Sec^ sequences and the same statistical approaches as mentioned above to determine deletion and duplication events. The constructed phylogenetic tree of tRNA^Sec^ genes was exported in a Newick file format. Subsequently, a species tree was constructed using all of the 128 species in the taxonomy browser of NCBI. To determine RNA^Sec^ deletion and duplication events, the phylogenetic tree of tRNA^Sec^ was reconciled with the species tree using Notung software, version 2.9. The reconciled gene and species tree revealed deletion, duplication, and co-divergence events that occurred in tRNA^Sec^ genes. The resultant phylogenetic tree of tRNAs (with tRNA^Sec^) and the phylogenetic tree of tRNA^Sec^ were analysed by using Icy Tree to identify recombination events.

### Cluster based grouping of the anti-codons

Anti-codons were grouped based on their percentage frequency in the tRNAnome. To cluster them, the percent frequency of anti-codons was used against each anti-codon. A classical clustering approach was used to cluster the anti-codons using a paired group UPGMA algorithm and Euclidean similarity index with 1000 bootstrap replicates.

### Statistical analysis

The probability plot linear regression analysis of tRNA gene number per genome and frequency of anti-codons were statistically analysed and a value of *p* < 0.05 was considered to be significant. To investigate anti-codon numbers in different lineages and their statistical significance, a t-test was conducted comparing anti-codon number in eudicot vs. monocot, eudicot vs. algae, and monocot vs. algae. Differences were deemed significant at *p* < 0.05. All of the statistical analyses were conducting using Past3 software.

## Results

### Genome size is not proportional to the number of tRNA genes

**Fig. 1:**
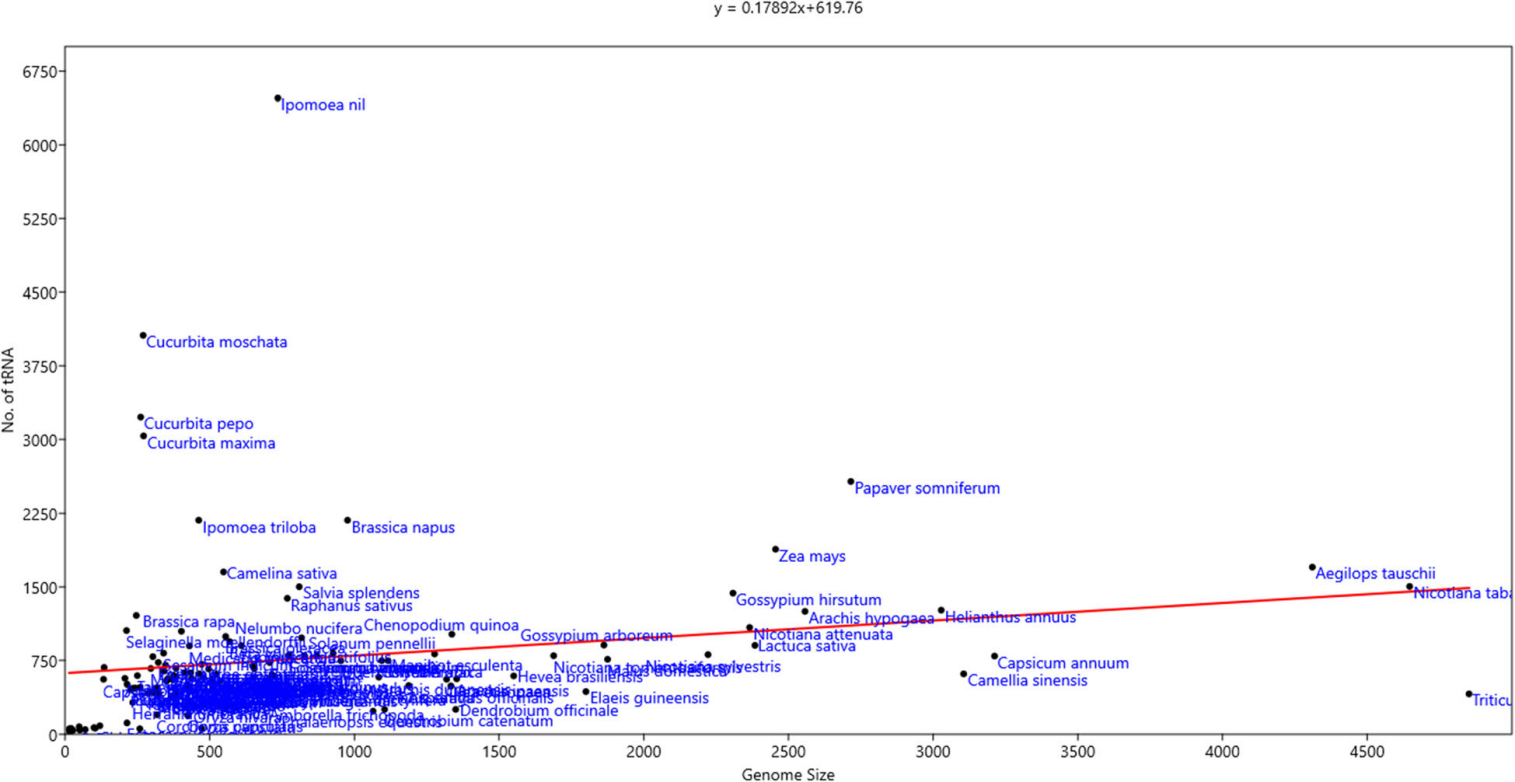
Regression analysis of tRNA gene number with plant genome. The analysis indicated that the number of tRNAs were not significantly correlated with plant genome size

A genome-wide analysis of fully-annotated whole genome sequences of 128 plant species was conducted to identify *tRNA* genes and to construct an anti-codon table of the plant kingdom (Table 1). The species included in the study varied in the size of their respective genomes (Table 2) A regression analysis was conducted to determine the correlation between genome size and the number of *tRNA* genes encoded per genome. Results indicated that plant genome size was not correlated (*r* = 0.5471, y = 0.17892x + 619.76) with the number of the *tRNA* genes per genome (Fig. 1). *Ipomoea nil,* with a genome size of genome size of 735.23 Mb, possesses 6475 *tRNA* genes which was the highest number of *tRNA* encoding genes identified in the species of plants that were analysed. Other species with a high number of *tRNA* genes in their genome were *Cucurbita moschata* (4062), *Cucurbita pepo* (3228), *Cucurbita maxima* (3036), *Papaver somniferum* (2571), *Brassica napus* (2180), and *Ipomoea triloba* (2180). Among the 128 analysed plant species, 22 (16.92%) species possessed more than 1 thousand *tRNA* genes in their genome. In contrast, *Ostreococcus tauri* and *Phaedactylum tricornutum* only encoded 41 tRNA genes in their genome, which was the lowest number of *tRNA* genes in the analysed genomes. Other species encoding lower number of *tRNA* genes were *Raphidocelis subcapitata* (43), *Monoraphidium neglectum* (48), and *Bathycoccus prasinus* (57). The genome size of *O. tauri, P. tricornutum, R. subcapitata, and M. neglectum* was 14.76, 27.4, 51.16, and 69.71 Mb, respectively. These genome sizes are relatively smaller than the genome of most of the other plant species that were analysed.

**Table 1:** Anti-codon table of the plant kingdom with frequency of anti-codons

tRNA Isotypes	Isoacceptors						Total no of Anti-codons (%)
Asparagine	AUU (155)	GUU (3972)					4.176
Cysteine	GCA (2454)	ACA (64)					2.547
Glutamine	CUG (1321)	UUG (1775)					3.133
Glycine	ACC (31)	GCC (3766)	CCC (1105)	UCC (2532)			7.523
Serine	GGA (378)	AGA (2332)	CGA (820)	UGA (1506)	ACU (19)	GCU (2522)	7.668
Threonine	AGU (1946)	GGU (366)	CGU (729)	UGU (1619)			4.716
Tyrosine	AUA (38)	GUA (2825)					2.897
Alanine	AGC (2897)	GGC (25)	CGC (1060)	UGC (2125)			6.180
Isoleucine	AAU (3200)	GAU (188)	UAU (1069)	CAU (0)			4.510
Leucine	AAG (2065)	GAG (9)	CAG (859)	UAG (1625)	CAA (2145)	UAA (1012)	7.808
Methionine	CAU (4979)						5.039
Phenylalanine	AAA (55)	GAA (3330)					3.425
Proline	AGG (2070)	GGG (22)	CGG (845)	UGG (2861)			5.868
Tryptophan	CCA (2736)						2.769
Valine	AAC (2700)	GAC (205)	CAC (1934)	UAC (1267)			6.179
Arginine	ACG (2178)	GCG (4)	CCG (705)	UCG (1084)	CCU (1311)	UCU (1915)	7.284
Histidine	AUG (53)	GUG (2031)					2.109
Lysine	CUU (3387)	UUU (2862)					6.324
Aspartic acid	GUC (4223)	AUC (52)					4.326
Glutamic acid	CUC (2909)	UUC (2370)					5.342
Suppressor	CUA (11)	UUA (25)					0.036
Selenocysteine	UCA (66)						0.066
Not determined	? (59)						0.059

**Table 2:** Genomic details of plant anti-codons

Species Name with Genome size (Mb)	Classification	Total No of tRNAs	Total No. of anti-codons	Missing anti-codons	Amino Acids of Missing anticodons	Missing tRNA Genes	Encoding Sec Amino Acids
*Abrus precatorius (347.23)*	Eudicot	702	49	ACA, AUG, AUA, ACC, GGC, UUA, GGG, ACU, CUA, GAG, GCG	His, Tyr, Cys, Val, Ala, Sup, Pro, Ser, Sup, Leu		
*Aegilops tauschii (4310.35)*	Monocot	1701	53	ACA, ACC, ACU, AUA, AUG, CUA, GAG, GCG, GGC, GGG, UUA	Cys, Gly, Ser, Tyr, His, Sup, Leu, Arg, Ala, Pro, Sup		Yes
*Amborella trichopoda (706.5)*	Amborella	321	49	AAA, ACA, ACC, ACU, AUA, AUC, AUG, AUU, CUA, GAG, GCG, GGC, GGG, UCA, UUA	Phe, Cys, Gly, Ser, Tyr, Asp, His, Asn, Sup, Leu, Arg, Ala, Pro, Sup, Sup		
*Ananas comosus (382.06)*	Monocot	446	49	AAA, ACA, ACC, ACU, AUA, AUC, AUG, AUU, CUA, GAG, GCG, GGC, GGG, UCA, UUA	Phe, Cys, Gly, Ser, Tyr, Asp, His, Asn, Sup, Leu, Arg, Ala, Pro, Sup, Sup		
*Arabidopsis thaliana (135)*	Eudicot	678	49	AAA, ACA, ACC, AUA, AUC, AUG, AUU, CUA, GAG, GCG, GGC, GGG, UCA, UUA	Phe, Cys, Gly, Tyr, Asp, His, Asn, Sup, Leu, Arg, Ala, Pro, Sup, Sup		
*Arachis duranensis (1084.26)*	Eudicot	579	51	AAA, ACA, ACC, ACU, AUA, CUA, GAC, GAG, GAU, GCG, GGG, UCA, UUA	Phe, Cys, Gly, Ser, Tyr, Sup, Val, Leu, Ile, Arg, Pro, Sup, Sup		
*Arachis hypogaea (2557.07)*	Eudicot	1250	56	ACC, CUA, GAG, GCG, GGC, GGG, UCA, UUA	Val, Sup, Leu, Arg, Ala, Pro, Sup, Sup		
*Arachis ipaensis (1353.5)*	Eudicot	562	52	AAA, ACC, ACU, AUG, CUA, GAC, GAG, GAU, GCG, GGC, UCA, UUA	Phe, Gly, Ser, His, Sup, Val, Leu, Ile, Arg, Ala, Sup, Sup		
*Arabidopsis lyrata (206.82)*	Eudicot	567	50	AAA, ACA, ACC, ACU, AUA, AUG, AUU, CUA, GAG, GCG, GGC, GGG, UCA, UUA	Phe, Cys, Gly, Ser, Tyr, His, Asn, Sup, Leu, Arg, Ala, Pro, Sup, Sup		
*Asparagus officinalis (1187.54)*	Monocot	493	45	AAA, ACA, ACC, AUA, AUC, AUG, AUU, CUA, GAC, GAG, GAU, GCG, GGA, GGC, GGG, GGU, UCA, UUA	Phe, Cys, Gly, Tyr, Asp, His, Asn, Sup, Val, Leu, Ile, Arg, Ser, Ala, Pro, Thr, Sup, Sup		
*Bathycoccus prasinos (15.07)*	Algae	57	31	AAA, AAG, AAU, ACA, ACC, ACU, AGA, AUA, AUC, AUG, AUU, CAC, CCC, CCG, CCU, CGA, CGC, CGU, CUA, CUC, CUG, GAC, GCC, GCG, GGA, GGC, GGG, GGU, UAU, UCA, UCG, UGU, UUA,	Phe, Leu, Ile, Cys, Gly, Ser, Ser, Tyr, Asp, His, Asn, Val, Gly, Arg, Arg, Ser, Ala, Thr, Sup, Glu, Gln, Val, Gly, Arg, Ser, Ala, Pro, Thr, Ile, Sup, Arg, Thr, Sup		
*Beta vulgaris (568.61)*	Eudicot	942	53	AAA, ACA, ACC, ACU, AUA, AUC, AUG, CUA, GAG, GCG, UUA	Phe, Cys, Gly, Ser, Tyr, Asp, His, Sup, Leu, Arg, Sup		Yes
*Brachypodium distachyon (355)*	Monocot	563	49	AAA, ACA, ACC, ACU, AUA, AUC, AUG, AUU, CUA, AGA, GCG, GGC, GGG, UCA, UUA	Phe, Cys, Gly, Ser, Tyr, Asp, His, Asn, Sup, Ser, Arg, Ala, Pro, Sup, Sup		
*Brassica napus (976.19)*	Eudicot	2180	53	AAA, ACA, ACC, ACU, AUA, AUU, CUA, GCG, GGG, UCA, UUA	Cys, Gly, Ser, Tyr, Asn, Sup, Arg, Pro, Sup, Sup, Phe		
*Brassica oleracea (554.98)*	Eudicot	993	48	AAA, ACA, ACC, ACU, AUC, AUG, CUA, GAC, GAG, GAU, GCG, GGC, GGG, GGU, UCA, UUA	Cys, Gly, Ser, Asp, His, Sup, Val, Leu, Ile, Arg, Ala, Pro, Thr, Sup, Sup, Phe		
*Brassica rapa (401.93)*	Eudicot	1047	50	AAA, ACA, ACC, ACU, AUA, AUC, AUG, AUU, CUA, GAG, GCG, GGA, GGG, UUA	Cys, Gly, Ser, Tyr, Asp, His, Asn, Sup, Leu, Arg, Ser, Pro, Sup, Phe		Yes
*Cajanus cajan (648.28)*	Eudicot	694	49	AAA, ACA, ACC, ACU, AUA, AUC, AUG, AUU, CUA, GAG, GCG, GGC, GGG, UCA, UUA	Cys, Gly, Ser, Tyr, Asp, His, Asn, Sup, Leu, Arg, Ala, Pro, Sup, Sup, Phe		
*Camelina sativa (547.65)*	Eudicot	1652	51	ACA, ACC, ACU, AUA, AUU, CUA, GAC, GAG, GAU, GCG, GGC, GGG, UCA, UUA	Gly, Ser, Tyr, Asn, Sup, Val, Leu, Ile, Arg, Ala, Pro, Sup, Sup, Cys		
*Camellia sinensis (3105.37)*	Eudicot	612	51	AAA, ACC, ACU, AUA, AUC, AUG, CUA, GAG, GCG, GGC, GGG, UCA, UUA	Gly, Ser, Tyr, Asp, His, Sup, Leu, Arg, Ala, Pro, Sup, Sup, Phe		
*Cannabis sativa (1333.38)*	Eudicot	490	46	AAA, ACA, ACC, ACU, AUA, AUC, AUG, AUU, CUA, GAC, GAG, GAU, GCG, GGA, GGC, GGG, UCA, UUA	Cys, Gly, Ser, Tyr, Asp, His, Asn, Sup, Val, Leu, Ile, Arg, Ser, Ala, Pro, Sup, Sup, Phe		
*Capsella rubella (133.06)*	Eudicot	557	47	AAA, ACA, ACC, ACU, AUA, AUC, AUG, AUU, CUA, GAC, GAG, GAU, GCG, GGC, GGG, UCA, UUA	Cys, Gly, Ser, Tyr, Asp, His, Asn, Sup, Val, Leu, Ile, Arg, Ala, Pro, Sup, Sup, Phe		
*Capsicum annuum (3212.12)*	Eudicot	794	53	ACA, ACC, ACU, AUA, AUC, CUA, GAG, GCG, GGC, UCA, UUA	Cys, Gly, Ser, Tyr, Asp, Sup, Leu, Arg, Ala, Sup, Sup		
*Carica papaya (370.42)*	Eudicot	378	51	ACA, ACC, ACU, AUA, AUC, AUG, CUA, GAG, GCG, GGC, GGG, UCA, UUA	Cys, Gly, Ser, Tyr, Asp, His, Sup, Leu, Arg, Ala, Pro, Sup, Sup		
*Chenopodium quinoa (1336.74)*	Eudicot	1017	51	AAA, ACC, ACU, AUA, AUC, AUG, CUA, GAG, GCG, GGC, GGG, UCA, UUA	Phe, Gly, Ser, Tyr, Asp, His, Sup, Leu, Arg, Ala, Pro, Sup, Sup		
*Chlamydomonas reinhardtii (120.41)*	Algae	87	45	AAA, ACA, ACC, ACU, AUA, AUC, AUG, AUU, CUA, GAC, GAG, GAU, GCG, GGA, GGC, GGG, GGU, UCA, UUA	Phe, Cys, Gly, Ser, Tyr, Asp, His, Asn, Sup, Val, Leu, Ile, Arg, Ser, Ala, Pro, Thr, Sup, Sup		
*Cicer arietinum (653.87)*	Eudicot	665	48	AAA, ACA, ACC, ACU, AUA, AUC, AUG, AUU, CUA, GAG, GCG, GGC, GGG, GGU, UCA, UUA	Phe, Cys, Gly, Ser, Tyr, Asp,His, Asn, Sup, Leu, Arg, Ala, Pro, Thr, Sup, Sup		
*Citrus clementina (301.37)*	Eudicot	428	47	AAA, ACA, ACC, ACU, AUA, AUC, AUG, AUU, CUA, GAC, GAG, GAU, GCG, GGC, GGG, UCA, UUA	Phe, Cys, Gly, Ser, Tyr, Asp, His, Asn, Sup, Val, Leu, Ile, Arg, Ala, Pro, Sup, Sup		
*Citrus sinensis (319.23)*	Eudicot	417	48	AAA, ACA, ACC, ACU, AUA, AUC, AUG, AUU, CUA, GAG, GAU, GCG, GGC, GGG, UCA, UUA	Phe, Cys, Gly, Ser, Tyr, Asp, His, Asn, Sup, Leu, Ile, Arg, Ala, Pro, Sup, Sup		
*Coccomyxa subellipsoidea (48.83)*	Algae	77	44	AAA, ACA, ACC, ACU, AUA, AUC, AUG, AUU, CUA, GAC, GAG, GAU, GCG, GGA, GGC, GGG, GGU, GUA, UCA, UUA	Phe, Cys, Gly, Ser, Tyr, Asp, His, Asn, Sup, Val, Leu, Ile, Arg, Ser, Ala, Pro, Thr, Tyr, Sup, Sup	Tyr	
*Coffea arabica (1094.45)*	Eudicot	747	50	AAA, ACA, ACU, AUA, AUC, AUG, CUA, GAG, GAU, GCG, GGC, GGG, UCA, UUA	Phe, Cys, Ser, Tyr, Asp, His, Sup, Leu, Ile, Arg, Ala, Pro, Sup, Sup		
*Coffea eugenioides (699.9)*	Eudicot	529	49	AAA, ACA, ACU, AUA, AUC, AUG, CUA, GAC, GAG, GAU, GCG, GGC, GGG, UCA, UUA	Phe, Cys, Ser, Tyr, Asp, His, Sup, Val, Leu, Ile, Arg, Ala, Pro, Sup, Sup		
*Corchorus capsularis (317.18)*	Eudicot	200	38	AAA, AAU, ACA, ACC, ACG, AGC, AGG, AUA, AUC, AUG, AUU, CAA, CGA, CGC, CUA, CUU, GAC, GAG, GAU, GCG, GGC, GGG, GUA, UCA, UUA, UUU	Phe, Ile, Cys, Gly, Arg, Ala, Pro, Tyr, Asp, His, Asn, Leu, Ser, Ala, Sup, Lys, Val, Leu, Ile, Arg, Ala, Pro, Tyr, Sup, Sup, Lys	Lys, Tyr	
*Corchorus olitorius (377.38)*	Eudicot	473	49	AAA, ACA, ACC, ACU, AUA, AUC, AUG, CUA, GAG, GAU, GCG, GGC, GGG, UCA, UUA	Phe, Cys, Gly, Ser, Tyr, Asp, His, Sup, Leu, Ile, Arg, Ala, Pro, Sup, Sup	Tyr	
*Cucumis melo (374.93)*	Eudicot	598	49	AAA, ACA, ACC, ACU, AUA, AUC, AUG, AUU, CUA, GAG, GCG, GGC, GGG, UCA, UUA	Phe, Cys, Gly, Ser, Tyr, Asp, His, Asn, Sup, Leu, Arg, Ala, Pro, Sup, Sup		
*Cucumis sativus (342.29)*	Eudicot	640	52	AAA, ACC, ACU, AUA, AUG, AUU, CUA, GAG, GCG, GGC, GGG, UUA	Phe, Gly, Ser, Tyr, His, Asn, Sup, Leu, Arg, Ala, Pro, Sup		Yes
*Cucurbita maxima (271.41)*	Eudicot	3036	55	ACU, AUA, AUG, CUA, GAG, GCG, GGA, GGC, UUA	Ser, Tyr, His, Sup, Leu, Arg, Ser, Ala, Sup		Yes
*Cucurbita moschata (269.94)*	Eudicot	4062	57	CUA, GAC, GAG, GCG, GGC, GGG, UUA	Sup, Val, Leu, Arg, Ala, Pro, Sup		Yes
*Cucurbita pepo (261.36)*	Eudicot	3228	56	ACC, AUC, AUG, CUA, GAG, GCG, GGG, UUA	Gly, Asp, His, Sup, Leu, Arg, Pro, Sup		Yes
*Cynara cardunculus (725.2)*	Eudicot	586	47	AAA, ACA, ACC, ACU, AUA, AUC, AUU, CUA, GAC, GAG, GAU, GCG, GGC, GGG, GGU, UCA, UUA	Phe, Cys, Gly, Ser, Tyr, Asp, Asn, Sup, Val, Leu, Ile, Arg, Ala, Pro, Thr, Val, Sup		
*Cyanophora paradoxa (100)*	Algae	68	47	AAA, ACA, ACC, ACU, AUA, AUC, AUG, AUU, CUA, GCG, GGA, GGC, GGG, GGU, UCA, UGA, UUA	Phe, Cys, Gly, Ser, Tyr, Asp, His, Asn, Sup, Arg, Ser, Ala, Pro, Thr, Sup, Ser, Sup		
*Daucus carota (421.54)*	Eudicot	494	49	AAA, ACA, ACC, ACU, AUC, AUG, AUU, CUA, GAG, GAU, GCG, GGC, GGG, UCA, UUA	Phe, Cys, Gly, Ser, Asp, His, Asn, Sup, Leu, Ile, Arg, Ala, Pro, Sup, Sup		
*Dendrobium catenatum (1104.26)*	Monocot	254	49	AAA, ACA, ACC, ACU, AUA, AUC, AUG, AUU, CUA, GAG, GCG, GGC, GGG, UCA, UUA	Phe, Cys, Gly, Ser, Tyr, Asp, His, Asn, Sup, Leu, Arg, Ala, Pro, Sup, Sup		
*Dendrobium officinale (1350)*	Orchid	254	49	AAA, ACA, ACC, ACU, AUA, AUC, AUG, AUU, CUA, GAG, GCG, GGC, GGG, UCA, UUA	Phe, Cys, Gly, Ser, Tyr, Asp, His, Asn, Sup, Leu, Arg, Ala, Pro, Sup, Sup		
*Durio zibethinus (715.23)*	Eudicot	604	48	AAA, ACA, ACC, ACU, AUA, AUC, AUG, AUU, CUA, GAC, GAG, GAU, GCG, GGG, UCA, UUA	Phe, Cys, Gly, Ser, Tyr, Asp, His, Asn, Sup, Val, Leu, Ile, Arg, Pro, Sup, Sup		
*Ectocarpus siliculosus (214)*	Algae	116	48	AAA, AAC, AAU, ACA, ACC, ACU, AUA, AUC, AUG, AUU, GAG, GCG, GGC, GGG, GGU, UUA	Phe, Val, Ile, Cys, Gly, Ser, Tyr, Asp, His, Asn, Leu, Arg, Ala, Pro, Thr		Yes, Sup
*Elaeis guineensis (1800)*	Monocot	436	49	AAA, ACA, ACC, ACU, AUA, AUC, AUG, AUU, CUA, GAG, GCG, GGC, GGG, UCA, UUA	Phe, Cys, Gly, Ser, Tyr, Asp, His, Asn, Sup, Leu, Arg, Ala, Pro, Sup, Sup		
*Erythranthe guttata (322.17)*	Eudicot	729	49	AAA, ACA, ACC, ACU, AUA, AUC, AUG, AUU, CUA, GAG, GCG, GGC, GGG, UCA, UUA	Phe, Cys, Gly, Ser, Tyr, Asp, His, Asn, Sup, Leu, Arg, Ala, Pro, Sup, Sup		
*Eucalyptus grandis (691.43)*	Eudicot	463	50	AAA, ACC, ACU, AUA, AUC, AUG, CUA, GAG, GCG, GGA, GGC, GGG, UCA, UUA	Phe, Gly, Ser, Tyr, Asp, His, Sup, Ser, Arg, Ser, Ala, Pro, Sup, Sup		
*Eutrema salsugineum (243.11)*	Eudicot	468	46	AAA, ACA, ACC, ACU, AUA, AUC, AUG, AUU, CUA, GAC, GAG, GAU, GCG, GGC, GGG, GGU, UCA, UUA	Phe, Cys, Gly, Ser, Tyr, Asp, His, Asn, Sup, Val, Leu, Ile, Arg, Ala, Pro, Thr, Sup, Sup		
*Fragaria vesca (214.37)*	Eudicot	507	49	AAA, ACA, ACC, ACU, AUA, AUC, AUG, AUU, CUA, GAG, GCG, GGC, GGG, UCA, UUA	Phe, Cys, Gly, Ser, Tyr, Asp, His, Asn, Sup, Leu, Arg, Ala, Pro, Sup, Sup		
*Glycine max (1116.18)*	Eudicot	747	50	AAA, ACA, ACC, ACU, AUA, AUC, AUG, CUA, GAG, GCG, GGC, GGG, UCA, UUA	Phe, Cys, Gly, Ser, Tyr, Asp, His, Sup, Leu, Arg, Ala, Pro, Sup, Sup		
*Gossypium arboreum (1862.24)*	Eudicot	906	50	AAA, ACA, ACC, ACU, AUA, AUC, AUG, CUA, GAG, GCG, GGC, GGG, UCA, UUA	Phe, Cys, Gly, Ser, Tyr, Asp, His, Sup, Leu, Arg, Ala, Pro, Sup, Sup		
*Gossypium hirsutum (2308.22)*	Eudicot	1436	55	ACC, ACU, AUA, CUA, GAG, GCG, GGC, UCA, UUA	Gly, Ser, Tyr, Sup, Leu, Arg, Ala, Sup, Sup		
*Gossypium raimondii (773.77)*	Eudicot	798	51	AAA, ACA, ACC, ACU, AUA, AUG, CUA, GAG, GCG, GGC, GGG, UCA, UUA	Phe, Cys, Gly, Ser, Tyr, His, Sup, Leu, Arg, Ala, Pro, Sup, Sup		
*Helianthus annuus (3027.84)*	Eudicot	1262	49	AAA, ACA, ACC, ACU, AUC, AUG, CUA, GAG, GAU, GCG, GGC, GGG, GGU, UCA, UUA	Phe, Cys, Gly, Ser, Asp, His, Sup, Leu, Ile, Arg, Ala, Pro, Thr, Sup, Sup		
*Herrania umbratica (234.04)*	Eudicot	325	45	AAA, ACA, ACC, ACU, AUA, AUC, AUG, AUU, CUA, GAC, GAG, GAU, GCG, GGA, GGC, GGG, GGU, UCA, UUA	Phe, Cys, Gly, Ser, Tyr, Asp, His, Asn, Sup, Val, Leu, Ile, Arg, Ser, Ala, Pro, Thr, Sup, Sup		
*Hevea brasiliensis (1550.51)*	Eudicot	592	49	AAA, ACA, ACC, ACU, AUA, AUC, AUG, AUU, CUA, GAG, GCG, GGC, GGG, UCA, UUA	Phe, Cys, Gly, Ser, Tyr, Asp, His, Asn, Sup, Leu, Arg, Ala, Pro, Sup, Sup		
*Ipomoea nil (735.23)*	Eudicot	6475	59	CUA, GAG, GGC, GGG, UUA	Sup, Leu, Ala, Pro, Sup		Yes
*Ipomoea triloba (461.99)*	Eudicot	2180	58	ACU, CUA, GAG, GCG, GGG, UUA	Ser, Sup, Leu, Arg, Pro, Sup		Yes
*Jatropha curcas (318.53)*	Eudicot	471	49	AAA, ACA, ACC, ACU, AUA, AUC, AUG, AUU, CUA, GAG, GCG, GGC, GGG, UCA, UUA	Phe, Cys, Gly, Ser, Tyr, Asp, His, Asn, Sup, Leu, Arg, Ala, Pro, Sup, Sup		
*Juglans regia (650.48)*	Eudicot	572	45	AAA, ACA, ACC, ACU, AUA, AUC, AUG, AUU, CUA, GAC, GAG, GAU, GCG, GGA, GGC, GGG, GGU, UCA, UUA	Phe, Cys, Gly, Ser, Tyr, Asp, His, Asn, Sup, Val, Leu, Ile, Arg, Ser, Ala, Pro, Thr, Sup, Sup		
*Klebsormidium nitens (104.21)*	Algae	62	29	AAA, AAC, AAG, AAU, ACA, ACC, ACU, AGA, AGC, AGG, AGU, AUA, AUC, AUG, AUU, CAC, CAG, CCC, CCG, CCU, GCG, CGG, CGU, CUA, CUC, CUG, CUU, GAC, GAG, GCG, GGC, UCA, UGA, UGU, UUA	Phe, Val, Leu, Ile, Cys, Gly, Ser, Ser, Ala, Pro, Thr, Tyr, Asp, His, Asn, Val, Leu, Gly, Arg, Ala, Pro, Thr, Sup, Glu, Gln, Lys, Val, Leu, Arg, Ala, Sup. Thr, Sup	Tyr, Ser	
*Lactuca sativa (2384.1)*	Eudicot	903	53	AAA, ACC, ACU, AUA, AUG, CUA, GAG, GCG, GGC, GGG, UUA	Phe, Gly, Ser, Tyr, His, Sup, Leu, Arg, Ala, Pro, Sup		Yes
*Lupinus angustifolius (609.2)*	Eudicot	900	47	AAA, ACA, ACU, AUA, AUC, AUG, AUU, CUA, GAC, GAG, GAU, GCG, GGC, GGG, GGU, UCA, UUA	Phe, Cys, Ser, Tyr, Asp, His, Asn, Sup, Val, Leu, Ile, Arg, Ala, Pro, Thr, Sup, Sup		
*Malus domestica (1874.77)*	Eudicot	761	47	AAA, ACA, ACC, ACU, AUA, AUC, AUG, CUA, GAC, GAG, GAU, GCG, GGA, GGC, GGU, UCA, UUA	Phe, Cys, Gly, Ser, Tyr, Asp, His, Sup, Val, Leu, Ile, Arg, Ala, Thr, Sup, Sup		
*Manihot esculenta (1276.89)*	Eudicot	815	50	ACA, ACC, ACU, AUA, AUC, AUG, AUU, CUA, GAG, GCG, GGC, GGG, UCA, UUA	Cys, Gly, Ser, Tyr, Asp, His, Asn, Sup, Leu, Arg, Ala, Sup, Sup		
*Medicago truncatula (429.61)*	Eudicot	898	55	ACU, AUC, AUG, CUA, GAG, GCG, GGC, GGG, UCA	Cys, Asp, His, Leu, Arg, Ala, Pro		Supr
*Micromonas commoda (21.11)*	Algae	60	26	AAA, AAC, AAG, AAU, ACA, ACC, ACU, AGA, AGC, AGG, AGU, AUA, AUC, AUG, AUU, CAA, CAC, CAG, CCC, CCG, CCU, CGA, CGC, CGG, CGU, CUA, CUC, CUG, CUU, GAC, GCG, GGA, GGC, GGG, UAU, UCA, UCG, UUA	Phe, Val, Leu, Ile, Cys, Gly, Ser, Ser, Ala, Pro, Thr, Tyr, Asp, His, Asn, Leu, Val, Leu, Gly, Arg, Arg, Ser, Ala, Pro, Thr, Sup, Glu, Ala, Lys, Val, Arg, Ser, Ala, Pro, Ile, Sup, Arg, Sup		
*Momordica charantia (296.26)*	Eudicot	667	49	ACA, ACU, AUA, AUC, AUG, CUA, GAC, GAG, GAU, GCG, GGA, GGC, GGG, GGU, UUA	Cys, Ser, Tyr, Asp, His, Sup, Val, Leu, Ile, Arg, Ser, Ala, Pro, Thr, Sup		Yes
*Monoraphidium neglectum (69.71)*	Algae	48	30	AAA, AAC, AAG, AAU, ACA, ACC, ACU, AGA, AGU, AUA, AUC, AUG, AUU, CAG, CCC, CCG, CCU, CGA, CGG, CGU, CUA, CUC, GAC, GAG, GCG, GGC, GGG, GGU, UAA, UCA, UCG, UGA, UGU, UUA	Phe, Val, Leu, Ile, Cys, Gly, Ser, Ser, Thr, Tyr, Asp, His, Asn, Leu, Gly, Arg, Arg, Ser, Pro, Thr, Sup, Glu, Val, Leu, Arg, Ala, Pro, Thr, Leu, Sup, Arg, Ser, Thr, Sup	Thr	
*Morus notabilis (320.38)*	Eudicot	392	47	AAA, ACA, ACC, ACU, AUA, AUC, AUU, CUA, GAC, GAG, GAU, GCG, GGA, GGC, GGG, UCA, UUA	Phe, Cys, Gly, Ser, Tyr, Asp, Asn, Sup, Val, Leu, Ile, Arg, Ser, Ala, Pro, Sup, Sup		
*Musa acuminata (472.23)*	Monocot	710	48	ACA, ACC, ACU, AUA, AUC, AUG, CUA, GAC, GAG, GAU, GCG, GGA, GGG, GGU, UCA, UUA	Cys, Gly, Ser, Tyr, Asp, His, Sup, Val, Leu, Ile, Arg, Ser, Pro, Thr, Sup, Sup		
*Nelumbo nucifera (817.27)*	Eudicot	980	53	ACC, AUA, AUC, AUG, CUA, GAG, GCG, GGC, GGG, UCA, UUA	Gly, Tyr, Asp, His, Sup, Leu, Arg, Ala, Pro, Sup, Sup		
*Nicotiana attenuata (2365.68)*	Eudicot	1086	53	ACC, ACU, AUA, CUA, GAC, GAG, GAU, GGC, GGG, UCA, UUA	Gly, Ser, Tyr, Sup, Val, Leu, Ile, Ala, Pro, Sup, Sup		
*Nicotiana sylvestris (2221.99)*	Eudicot	809	52	AAA, ACC, ACU, AUA, AUC, AUU, GAG, GCG, GGC, GGG, UCA, UUA	Phe, Gly, Ser, Tyr, Asp, Asn, Leu, Arg, Ala, Pro, Sup, Sup		Supre
*Nicotiana tabacum (4646.65)*	Eudicot	1504	55	AAA, ACU, AUA, CUA, GAG, GCG, GGC, GGG, UUA	Phe, Ser, Tyr, Sup, Leu, Arg, Ala, Pro, Sup		Yes
*Nicotiana tomentosiformis (1688.47)*	Eudicot	798	52	AAA, ACC, ACU, AUC, AUG, CUA, GAG, GCG, GGC, GGG, UCA, UUA	Phe, Gly, Ser, Asp, His, Sup, Leu, Arg, Ala, Pro, Sup, Sup		
*Olea europaea (1318.65)*	Eudicot	557	52	AAA, ACC, AUC, AUG, CUA, GAG, GCG, GGA, GGC, GGG, UCA, UUA	Phe, Gly, Asp, His, Sup, Leu, Arg, Ser, Ala, Pro, Sup, Sup		
*Oryza barthii (308.27)*	Monocot	455	48	AAA, ACA, ACC, ACU, AUA, AUC, AUG, AUU, CUA, GAG, GAU, GCG, GGG, GGU, UCA, UUA	Phe, Cys, Gly, Ser, Tyr, Asp, Hus, Asn, Sup, Leu, Ile, Arg, Pro, Thr, Sup, Sup		
*Oryza brachyantha (259.91)*	Monocot	476	47	AAA, ACA, ACC, ACU, AUA, AUG, AUU, CUA, GAC, GAU, GCG, GGA, GGC, GGG, GGU, UCA, UUA	Phe, Cys, Gly, Ser, Tyr, His, Asn, Sup, Val, Ile, Arg, Ser, Ala, Pro, Thr, Sup, Sup		
*Oryza glaberrima (303.3)*	Monocot	788	48	AAA, ACA, ACC, ACU, AUA, AUC, AUG, AUU, CUA, GAG, GAU, GCG, GGC, GGG, UCA, UUA	Phe, Cys, Gly, Ser, Tyr, Asp, His, Asn, Sup, Leu, Ile, Arg, Ala, Pro, Sup, Sup		
*Oryza glumaepatula (372.86)*	Monocot	554	50	AAA, ACA, ACC, ACU, AUC, AUG, AUU, CUA, GAG, GAU, GCG, GGG, UCA, UUA	Phe, Cys, Gly, Ser, Asp, His, Asn, Sup, Leu, Ile, Arg, Pro, Sup, Sup		
*Oryza meridionalis (354.61)*	Monocot	552	48	AAA, ACA, ACC, ACU, AUA, AUC, AUG, AUU, CUA, GAG, GAU, GCG, GGC, GGG, UCA, UUA	Phe, Cys, Gly, Ser, Tyr, Asp, His, Asn, Sup, Leu, Ile, Arg, Ala, Pro, Sup, Sup		
*Oryza nivara (448)*	Monocot	290	48	AAA, ACA, ACC, ACU, AUC, AUG, AUU, CUA, GAG, GCG, GGA, GGC, GGG, GGU, UCA, UUA	Phe, Cys, Gly, Ser, Asp, His, Asn, Sup, Leu, Arg, Ser, Ala, Pro, Thr, Sup, Sup		
*Oryza punctata (423)*	Monocot	195	43	AAA, ACA, ACC, ACU, AUA, AUC, AUG, AUU, CUA, GAC, GAG, GAU, GCG, GGA, GGC, GGG, UAA, UAC, UAU, UCA, UUA	Phe, Cys, Gly, Ser, Tyr, Asp, His, Asn, Sup, Val, Leu, Ile, Arg, Ser, Ala, Pro, Leu, Val, Ile, Sup, Sup		
*Oryza rufipogon (384.52)*	Monocot	582	50	AAA, ACA, ACC, ACU, AUC, AUG, AUU, CUA, GAG, GAU, GCG, GGG, UCA, UUA	Phe, Cys, Gly, Ser, Asp, His, Asn, Sup, Leu, Ile, Arg, Pro, Sup, Sup		
*Oryza sativa (383.24)*	Monocot	668	51	AAA, ACC, ACU, AUA, AUG, AUU, CUA, GAG, GCG, GGC, GGG, UCA, UUA	Phe, Gly, Ser, Tyr, His, Asn, Sup, Ser, Arg, Ala, Pro, Sup, Sup		
*Ostreococcus tauri (14.76)*	Algae	41	18	AAA, AAG, AAU, ACA, ACC, ACG, ACU, AGG, AGU, AUA, AUC, AUG, AUU, CAA, CAC, CCC, CCG, CCU, CGA, CGC, CGG, CGU, CUA, CUC, CUG, CUU, GAA, GAC, GAG, GAU, GCG, GCU, GGA, GGC, GGG, GGU, UAA, UAC, UAG, UCA, UCC, UCU, UGA, UGC, UUA, UUG	Phe, Leu, Ile, Cys, Gly, Arg, Ser, Pro, Thr, Tyr, Asp, His, Asn, Leu, Val, Gly, Arg, Arg, Ser, Ala, Pro, Thr, Sup, Glu, Gln, Lys, Phe, Val, Leu, Ile, Arag, Ser, Ser, Ala, Pro, Thr, Leu, Val, Leu, Sup, Gly, Arg, Ser, Ala, Sup, Gln	Phe, Gln	
*Panicum hallii (535.89)*	Monocot	541	48	AAA, ACA, ACC, ACU, AUA, AUC, AUU, CUA, GAC, GAG, GAU, GCG, GGC, GGG, UCA, UUA	Phe, Cys, Gly, Ser, Tyr, Asp, Asn, Sup, Val, Leu, Ile, Arg, Ala, Pro, Sup, Sup		
*Papaver somniferum (2715.53)*	Eudicot	2571	59	AAA, ACU, CUA, GAG, UUA	Phe, Ser, Sup, Leu, Sup		Yes
*Picea glauca (258.27)*	Gymnosperm	57	30	AAA, AAC, AAG, AAU, ACA, ACC, ACU, AGA, AGC, AGG, AGU, AUA, AUC, AUG, AUU, CAC, CAG, CCC, CCU, CGA, CGC, CGG, CGU, CUA, CUC, CUG, CUU, GAG, GCG, GGA, GGC, UAA, UAU, UUA	Phe, Val, Leu, Ile, Cys, Gly, Ser, Ser, Ala, Pro, Thr, Tyr, Asp, His, Asn, Val, Leu, Gly, Arg, Ser, Ala, Pro, Thr, Sup, Glu, Gln, Lys, Leu, Arg, Ser, Ala, Leu, Ile, Sup	Ser	Yes
*Phaeodactylum tricornutum (27.4)*	Alage	41	38	AAA, ACA, ACC, ACG, ACU, AUA, AUC, AUG, AUU, CCC, CGG, CUA, GAC, GAG, GAU, GCA, GCG, GGA, GGC, GGG, GGU, UAU, UCA, UCU, UGG, UAA	Phe, Cys, Gly, Arg, Ser, Tyr, Asp, His, Asn, Gly, Pro, Sup, Val, Leu, Ile, Cys, Arg, Ser, Ala, Pro, Thr, Ile, Sup, Arg, Pro, Leu	Cys	
*Phalaenopsis equestris (1064.2)*	Monocot	236	48	AAA, ACA, ACC, ACU, AUA, AUC, AUG, AUU, CUA, GAG, GAU, GCG, GGC, GGG, UCA, UUA	Phe, Cys, Gly, Ser, Tyr, Asp, His, Asn, Sup, Leu, Ile, Arg, Ala, Pro, Sup, Sup		
*Phoenix dactylifera (854.66)*	Monocot	470	50	AAA, ACC, ACU, AUA, AUC, AUG, AUU, CUA, GAG, GCG, GGC, GGG, UCA, UUA	Phe, Gly, Ser, Tyr, Asp, His, Asn, Sup, Leu, Arg, Ala, Pro, Sup, Sup		
*Physcomitrella patens (472.08)*	Bryophyte	60	29	AAA, AAC, AAG, AAU, ACA, ACC, ACU, AGA, AGC, AGG, AGU, AUA, AUC, AUG, AUU, CAC, CAG, CCC, CCU, CGA, CGC, CGG, CGU, CUA, CUC, CUG, CUU, GAG, GCG, GGC, GGG, UAU, UCA, UCG, UUA	Phe, Val, Leu, Ile, Cys, Gly, Ser, Ser, Ala, Pro, Thr, Tyr, Asp, His, Asn, Val, Leu, Gly, Arg, Ser, Ala, Pro, Thr, Sup, Glu, Gln, Lys, Leu, Arg, Ala, Pro, Ile, Sup, Arg, Sup		
*Populus trichocarpa (434.29)*	Eudicot	623	49	AAA, ACA, ACC, ACU, AUA, AUC, AUG, AUU, CUA, GAG, GCG, GGC, GGG, UCA, UUA	Phe, Cys, Gly, Ser, Tyr, Asp, His, Asn, Sup, Leu, Arg, Ala, Pro, Sup, Sup		
*Populus euphratica (496.03)*	Eudicot	662	54	AAA, ACC, ACU, AUA, AUG, AUU, CUA, GAG, GCG, GGG	Phe, Gly, Ser, Tyr, His, Asn, Leu, Arg, Pro		Yes/Sup
*Prosopis alba (707.16)*	Eudicot	728	47	AAA, ACA, ACC, ACU, AUA, AUC, AUG, AUU, CUA, GAC, GAG, GAU, GCG, GGC, GGG, UCA, UUA	Phe, Cys, Gly, Ser, Tyr, Asp, His, Asn, Sup, Val, Leu, Ile, Arg, Ala, Pro, Sup, Sup		
*Prunus avium (287.19)*	Eudicot	419	46	AAA, ACA, ACC, ACU, AUA, AUG, AUU, CUA, GAC, GAG, GAU, GCG, GGA, GGC, GGG, GGU, UCA, UUA	Phe, Cys, Gly, Ser, Tyr, His, Asn, Sup, Val, Leu, Ile, Arg, Ser, Ala, Pro, Thr, Sup, Sup		
*Prunus dulcis (246.12)*	Eudicot	1208	49	AAA, ACA, ACC, ACU, AUC, AUG, AUU, CUA, GAG, GAU, GCG, GGC, GGG, UCA, UUA	Phe, Cys, Gly, Ser, His, Asn, Sup, Leu, Ile, Arg, Ala, Pro, Sup, Sup		
*Prunus mume (234.03)*	Eudicot	466	49	AAA, ACA, ACC, ACU, AUA, AUC, AUG, AUU, CUA, GAG, GCG, GGC, GGG, UCA, UUA	Phe, Cys, Gly, Ser, Asp, His, Asn, Sup, Leu, Arg, Ala, Pro, Sup, Sup		
*Prunus persica (227.57)*	Eudicot	452	49	AAA, ACA, ACC, ACU, AUA, AUC, AUG, AUU, CUA, GAG, GCG, GGC, GGG, UCA, UUA	Phe, Cys, Gly, Ser, Tyr, Asp, His, Asn, Sup, Leu, Arg, Ala, Pro, Sup, Sup		
*Punica granatum (380.18)*	Eudicot	430	49	AAA, ACA, ACC, ACU, AUA, AUC, AUG, AUU, CUA, GAG, GCG, GGC, GGG, UCA, UUA	Phe, Cys, Gly, Ser, Tyr, Asp, His, Asn, Sup, Leu, Arg, Ala, Pro, Sup, Sup		
*Pyrus bretschneideri (508.55)*	Eudicot	553	46	AAA, ACA, ACC, ACU, AUA, AUC, AUG, AUU, CUA, GAG, GAU, GCG, GGA, GGC, GGG, GGU, UCA, UUA	Phe, Cys, Gly, Ser, Tyr, Asp, His, Asn, Sup, Leu, Ile, Arg, Ser, Ala, Pro, Thr, Sup, Sup		
*Quercus suber (953.3)*	Eudicot	746	46	AAA, ACA, ACC, ACU, AUA, AUC, AUG, AUU, CUA, GAC, GAG, GAU, GCG, GGC, GGG, GGU, UCA, UUA	Phe, Cys, Gly, Ser, Tyr, Asp, His, Asn, Sup, Val, Leu, Ile, Arg, Ala, Pro, Thr, Sup, Sup		
*Raphanus sativus (768.11)*	Eudicot	1383	49	AAA, ACA, ACC, ACU, AUA, AUC, AUG, AUU, CUA, GAG, GCG, GGC, GGG, UCA, UUA	Phe, Cys, Gly, Ser, Tyr, Asp, His, Asn, Sup, Leu, Arg, Ala, Pro, Sup, Sup		
*Raphidocelis subcapitata (51.16)*	Algae	43	32	AAA, AAG, AAU, ACA, ACC, ACU, AUA, AUC, AUG, AUU, CUA, GAC, GAG, GAU, GCG, GGA, GGC, GGG, GGU, GUA, UAA, UAC, UAG, UCA, UGA, UGC, UGG, UGU, UUA, UUC, UUG, UUU	Phe, Leu, Ile, Cys, Gly, Ser, Tyr, Asp, His, Asn, Sup, Val, Leu, Ile, Arg, Ser, Ala, Pro, Thr, Tyr, Leu, Val, Leu, Sup, Ser, Ala, Pro, Thr, Sup, Glu, Gln, Lys	Tyr	
*Rhodamnia argentea (414.82)*	Eudicot	628	48	AAA, ACA, ACC, ACU, AUA, AUC, AUG, CUA, GAC, GAG, GAU, GCG, GGC, GGG, UCA, UUA	Phe, Cys, Gly, Ser, Tyr, Asp, His, Sup, Val, Leu, Ile, Arg, Ala, Pro, Sup, Sup		
*Ricinus communis (350.62)*	Eudicot	539	50	AAA, ACA, ACC, ACU, AUA, AUC, AUG, CUA, GAG, GCG, GGC, GGG, UCA, UUA	Phe, Cys, Gly, Ser, Tyr, Asp, His, Sup, Leu, Arg, Ala, Pro, Sup, Sup		
*Rosa chinensis (513.85)*	Eudicot	486	48	AAA, ACA, ACC, ACU, AUA, CUA, GAC, GAG, GAU, GCG, GGA, GGC, GGG, GGU, UCA, UUA	Phe, Cys, Gly, Ser, Tyr, Asp, His, Sup, Ile, Arg, Ser, Ala, Pro, Thr, Sup, Sup		
*Salvia splendens (809.16)*	Eudicot	1501	55	ACC, ACU, AUA, AUG, AUU, CUA, GAU, GCG, UUA	Gly, Ser, Tyr, His, Asn, Sup, Ile, Arg, Sup		Yes
*Selaginella moellendorffii (212.31)*	Pteridophyte	1054	48	AAA, ACC, AUA, AUC, AUG, AUU, CUA, GAC, GAG, GCG, GGA, GGC, GGG, GGU, UCA, UUA	Phe, Gly, Tyr, Asp, His, Asn, Sup, Val, Leu, Arg, Ser, Ala, Pro, Thr, Sup, Sup		
*Sesamum indicum (340.46)*	Eudicot	824	50	AAA, ACA, ACC, ACU, AUA, AUC, AUG, CUA, GAG, GCG, GGC, GGG, UCA, UUA	Phe, Cys, Gly, Ser, Tyr, Asp, His, Sup, Leu, Arg, Ala, Pro, Sup, Sup		
*Setaria italica (477.54)*	Monocot	576	49	AAA, ACA, ACC, ACU, AUA, AUC, AUG, AUU, CUA, GAG, GCG, GGC, GGG, UCA, UUA	Phe, Cys, Gly, Ser, Tyr, Asp, His, Asn, Sup, Leu, Arg, Ala, Pro, Sup, Sup		
*Solanum lycopersicum (828.35)*	Eudicot	793	48	AAA, ACA, ACC, ACU, AUA, AUC, AUG, AUU, CUA, GAG, GAU, GCG, GGC, GGG, UCA, UUA	Phe, Cys, Gly, Ser, Tyr, Asp, His, Asn, Sup, Leu, Ile, Arg, Ala, Pro, Sup, Sup		
*Solanum pennellii (926.43)*	Eudicot	823	49	AAA, ACA, ACC, AUA, AUC, AUU, CUA, GAC, GAG, GAU, GCG, GGC, GGG, UCA, UUA	Phe, Cys, Gly, Tyr, Asp, Asn, Sup, Val, Leu, Ile, Arg, Ala, Pro, Sup, Sup		
*Solanum tuberosum (772.25)*	Eudicot	799	50	AAA, ACA, ACC, ACU, AUA, AUC, CUA, GAG, GAU, GCG, GGC, GGG, UCA, UUA	Phe, Cys, Gly, Ser, Tyr, Asp, Sup, Leu, Ile, Arg, Ala, Pro, Sup, Sup		
*Sorghum bicolor (709.35)*	Monocot	550	49	AAA, ACA, ACC, ACU, AUA, AUC, AUU, CUA, GAG, GAU, GCG, GGC, GGG, UCA, UUA	Phe, Cys, Gly, Ser, Tyr, Asp, Asn, Sup, Leu, Ile, Arg, Ala, Pro, Sup, Sup		
*Spinacia oleracea (869.95)*	Eudicot	798	50	AAA, ACA, ACC, ACU, AUA, AUC, AUG, CUA, GAG, GCG, GGC, GGG, UCA, UUA	Phe, Cys, Gly, Ser, Tyr, Asp, His, Sup, Leu, Arg, Ala, Pro, Sup, Sup		
*Syzygium oleosum (431.29)*	Eudicot	445	47	AAA, ACA, ACC, ACU, AUA, AUC, AUG, AUU, CUA, GAC, GAG, GAU, GCG, GGC, GGG, UCA, UUA	Phe, Cys, Gly, Ser, Tyr, Asp, His, Asn, Sup, Val, Leu, Ile, Arg, Ala, Pro, Sup, Sup		
*Tarenaya hassleriana (249.93)*	Eudicot	595	46	AAA, ACA, ACC, ACU, AUA, AUC, AUG, AUU, CUA, GAC, GAG, GAU, GCG, GGA, GGC, GGG, GGU, UUA	Phe, Cys, Gly, Ser, Tyr, Asp, His, Asn, Sup, Val, Leu, Arg, Ala, Pro, Thr, Sup		Yes
*Theobroma cacao (324.88)*	Eudicot	415	49	AAA, ACA, ACC, ACU, AUA, AUC, AUG, AUU, CUA, GAG, GCG, GGC, GGG, UCA, UUA	Phe, Cys, Gly, Ser, Tyr, Asp, His, Asn, Sup, Arg, Ala, Pro, Sec, Sup		
*Triticum urartu (4851.9)*	Monocot	412	51	AAA, ACA, ACC, ACU, AUA, AUC, AUG, CCC, CUA, GCG, GGC, UAU, UUA	Phe, Cys, Gly, Ser, Tyr, Asp, His, Gly, Sup, Arg, Ala, Ile, Sup		Yes
*Vigna angularis (522.76)*	Eudicot	610	50	AAA, ACA, ACC, ACU, AUA, AUC, AUG, CUA, GAG, GCG, GGC, GGG, UCA, UUA	Phe, Cys, Gly, Ser, Tyr, Asp, His, Sup, Leu, Arg, Ala, Pro, Sup, Sup		
*Vigna radiata (463.64)*	Eudicot	614	50	ACA, ACC, ACU, AUA, AUC, AUG, AUU, CUA, GAG, GCG, GGC, GGG, UCA, UUA	Cys, Gly, Ser, Tyr, Asp, His, Asn, Sup, Leu, Arg, Ala, Pro, Sup, Sup		
*Vitis vinifera (868.04)*	Eudicot	448	50	AAA, ACA, ACC, ACU, AUA, AUG, AUU, CUA, GAG, GCG, GGC, GGG, UCA, UUA	Phe, Cys, Gly, Ser, Tyr, His, Asn, Sup, Leu, Arg, Ala, Pro, Sup, Sup	NA	
*Zea mays (2455.26)*	Monocot	1884	59	ACC, GAG, GAU, GCG, GGC	Gly, Leu, Ile, Arg, Ala	NA	Yes/Sup
*Ziziphus jujuba (437.75)*	Eudicot	566	52	ACC, ACU, AUA, AUC, AUG, CUA, GAC, GAG, GGC, GGG, UCA, UUA	Gly, Ser, Tyr, Asp, His, Sup, Val, Leu, Ala, Pro, Sup, Sup	NA

### CAU (met) was the most abundant and GCG (Arg) was the least abundant encoded anti-codons in the plant kingdom

The occurrence of each of the anti-codons were separately analysed to determine the frequency of anti-codons in the genomes of the Plant Kingdom. Results indicated that CAU (Met) was the most abundant (5.033%) anti-codon in the Plant Kingdom, followed by GUC (Asp, 4.274%), GUU (Asn, 4.020%), and GCC (Gly, 3.811%) (Table 1, Supplementary File 1). In contrast, GCG (Arg) was identified as the least abundant (0.004%) anti-codon in the Plant Kingdom, followed by GAG (Leu, 0.009%), CUA (Sup, 0.0111%), and ACU (Ser, 0.019%) (Table 1, Supplementary File 1). The lowest-abundant anti-codon (GCG) was only present in *Ipomea nil, Nicotiana attenuata, Papaver somniferum*, and *Ziziphus jujuba*. When the anti-codon frequency of different tRNA isoacceptor was considered, however, tRNA^Leu^ was found to be the most abundant tRNA isoacceptor (Table 1). Approximately 7.808% of all anti-codons in the Plant Kingdom were found to be encoded by tRNA^Leu^ (Table 1). The abundance of tRNA^Leu^, was followed by tRNA^Ser^ (7.668%), tRNA^Gly^ (7.523%), and tRNA^Arg^ (7.284%) (Table 1). tRNA^Leuc^, tRNA^Ser^, and tRNA^Arg^ encode six different isoacceptors which might be the reason for their higher abundance in the plant genomes. Suppressor tRNA (0.036%) was found to be the least abundant tRNA isoacceptor in the plant genomes, followed by tRNA^Sec^ (0.066%), tRNA^His^ (2.109%), and tRNA^Cys^ (2.547%) (Table 1). Suppressor tRNA (CUA) anti-codon was only found in *Ectocarpus siliculosus, Nicotiana sylvestris*, and *Zea mays* (Supplementary File 1).

### Anti-codons can be classified into five groups based on their frequency of occurrence in plant genomes

**Fig. 2:**
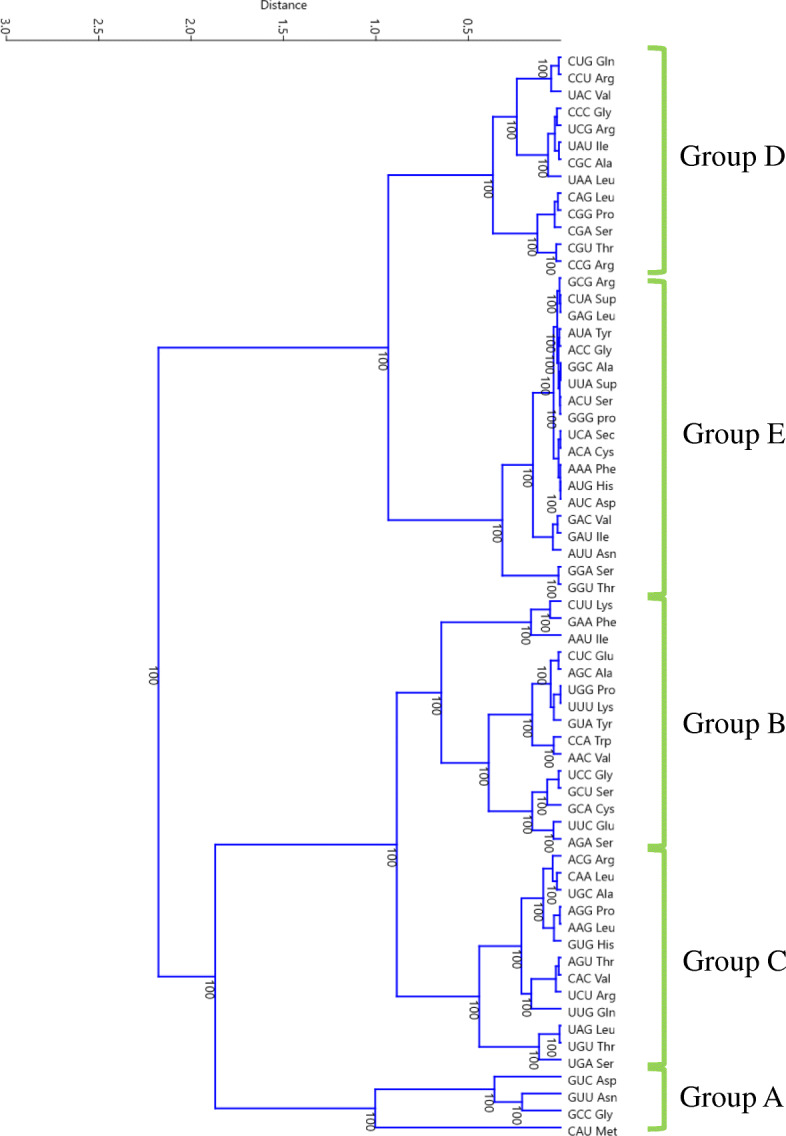
Grouping of plant anti-codons. The clustering was conducted based on the frequency (percentage) of the anti-codons found in the collective plant genomes of 128 plant species. The grouping A, B, C, D, and E were made based on the decreasing order of anti-codon frequency. The clustering was conducted using the UPGMA algorithm and Euclidean distance matrix with 1000 bootstrap replicates in Past3 software

A clustering analysis based on the frequency of abundance of the anti-codons in the Plant Kingdom was conducted using the paired group (UPGMA) algorithm and Euclidean similarity index with 1000 bootstrap replicates. The analysis revealed five distinct groups of anti-codons and were named as group A, B, C, D, and E (Fig. 2). The anti-codons in the different groups were: Group A - CAU, GCC, GUU, and GUC); Group B - CUU, GAA, AAU, AGA, UCC, GCA, GCU, UCC, AAC, CCA, GUA, UUU, UGG, AGC, UUC, and CUC; Group C - UGA, UGU, UAG, UUG, UCU, CAC, AGU, GUG, AAG, AGG, UGC, CAA, and ACG; Group D - CCG, CGU, CGA, CGG, CAG, UAA, CGC, UAU, UCG, CCC, UAC, CCU, and CUG; and Group E - GGU, GGA, AUU, GAU, GAC, AUC, AUG, AAA, ACA, UCA, GGG, ACU, UUA, GGC, ACC, AUA, GAG, CUA, and GCG (Fig. 2). The anti-codon groupings are based on their abundance in plant genomes, from highest (Group A) to lowest (Group E).

### Plant genomes encode 18 to 59 isoacceptors (anti-codons)

The genome-wide analysis of the Plant Kingdom revealed the diversity in the number of anti-codons present in the genomes of individual species, which ranged from 18 to 59 (Table 2). *Ostreococcus tauri* was found to encode only 18 isoacceptors while *Micromonas commoda* encodes only 26 isoacceptors (Table 2). *Ipomoea nil, Papaver somniferum*, and *Zea mays* encoded the highest number of anti-codons at 59 each. At least 51 (39.53%) species were found to encode 50 or more anti-codons in their genome. On average, plant genomes encode 48.25 anti-codons per genome. A paired two tailed t-test was conducted to statistically analyse the frequency of anti-codons present in algae, eudicot, and monocot species. The comparison between eudicot and monocot species indicated that the frequency of tRNA anti-codons in these two groups was not significantly different (*P* < 0.05) at 1.2691 < 1.984 (t-test result 1.2691, critical value *T* 1.984), respectively (Table 3). In contrast, a significant difference in tRNA frequency was observed between eudicots and algae (10.3939 > 1.987), and between monocots and algae (6.2914 > 2.037) (Table 3). Notably, the variance in tRNA frequency in the monocot lineage was much lower than it was in the eudicots and algae.

**Table 3:** *t-test* (two tailed) between eudicot and monocot anti-codon numbers. The *t-value* is smaller than critical value (1.2691 < 1.984). So, the mean was not significantly different (*p < 0.05*). (B) *t-test* (two tailed) between eudicot and algae anti-codon numbers. The t-test result was greater than critical value (10.3939 > 1.987). So, the mean is significantly different (*p < 0.05*). (C) t-test (two tailed) between Eudicot and algae anti-codon numbers. The t-test result was greater than critical value (6.2914 > 2.037). So, the mean is significantly different (*p < 0.05*)

(A)					
**Statistical parameters**	**Eudicot**	**Monocot**	***t***	**Degree of freedom**	**Critical value (*T*)**
Mean	50.0562	49.087	1.2691	110	1.984
Variance	11.1673	8.6285			
Stand. Dev	3.3418	2.9374			
n	89	23			
(B)					
**Statistical parameters**	**Monocot**	**Algae**	***t***	**Degree of freedom**	**Critical value (*T*)**
Mean	49.087	35.2727	6.2914	32	2.037
Variance	8.6285	95.8182			
Stand. Dev	2.9374	9.7878			
n	23	11			
(C)					
**Statistical parameters**	**Monocot**	**Algae**	***t***	**Degree of freedom**	**Critical value (*T*)**
Mean	49.087	35.2727	6.2914	32	2.037
Variance	8.6285	95.8182			
Stand. Dev	2.9374	9.7878			
n	23	11			

### Only a few species have lost tRNA genes

Our analysis revealed that a few species have lost the presence of specific tRNA genes (tRNA isotype) in their genome. These species include *Coccomyxa subellipsoidea* (tRNA^Tyr^), *Corchorus capsularis* (tRNA^Lys^, tRNA^Tyr^), *Corchorus olitorius* (tRNA^Tyr^), *Klebsormidium nitens* (tRNA^Tyr^, tRNA^Ser^), *Monoraphidium neglectum* (tRNA^Thr^), *Ostreococcus tauri* (tRNA^Phe^, tRNA^Gln^), *Picea glauca* (tRNA^Ser^), *Phaedactylum tricornutum* (tRNA^Cys^), and *Raphidocelis subcapitata* (tRNA^Tyr^) (Table 2). These species were found to lost the mentioned gene(s) in their genome. Understanding the loss of tRNA genes and its functional implication in protein translation is very crucial.

### Some plant species encode tRNA^Sec^ in their genomes

Several plant species were found to encode *tRNA* genes for selenocysteine amino acids. More specifically, 22 (17.187%) species were found to encode a *tRNA*^*Sec*^ gene in their genome. These species were *Aegilops tauschii, Beta vulgaris, Brassica rapa, Cucumis sativus, Cucurbita maxima, Cucurbita moschata, Cucurbita pepo, Ectocarpus siliculosus, Ipomoea nil, Ipomoea triloba, Lactuca sativa, Momordica charantia, Medicago truncatula, Monoraphidium neglectum, Nicotiana tabacum, Papaver somniferum, Picea glauca, Populus euphratica, Salvia splendens, Tarenaya hassleriana, Triticum urartu*, and *Zea mays* (Table 2). The length of *tRNA*^*Sec*^ encoding genes was ranged from 70 to 90 nucleotides with average length being 72.93 nucleotides per tRNA. A multiple sequence alignment of *tRNA*^*Sec*^ genes indicated the presence of a conserved G-x-C nucleotide at the 30th and 32nd positions and a conserved U-C-A at 34th, 35th, and 36th positions (Supplementary Figure 1). The pseudo-uridine loop was also found to contain a conserved G-U-U-x_2_-A-x_2_-C nucleotide consensus sequence (Supplementary Figure 1). The tRNA^Sec^ in *C. maxima* (NW_019272053.1), however, was found to encode a C-U-U nucleotide sequence instead of a G-U-U conserved consensus sequence in its pseudo-uridine loop (Supplementary Figure 1).

### Loss of tRNA^Sec^ occurred to a greater extent than duplication

**Fig. 3:**
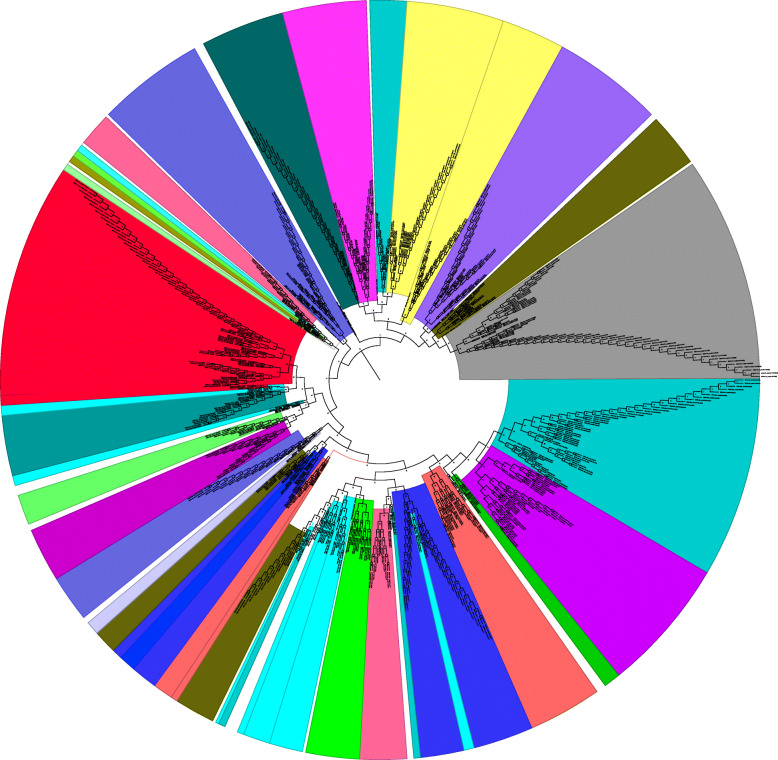
Phylogenetic tree of tRNA^Sec^ and other tRNA isotypes. The phylogenetic tree with 21 tRNA isotypes revealed at least 28 major phylogenetic groups where tRNA^Sec^ (red) was placed with different tRNA isotypes. The phylogenetic tree indicates that tRNA has most likely evolved from multiple common ancestors and has also undergone duplication. The evolutionary history was inferred using the Maximum Likelihood method based on the Kimura 2-parameter model. The tree with the highest log likelihood (− 7466.51) is illustrated. The percentage of the branches in which the associated taxa cluster together is shown next to the branches. Initial tree(s) for the heuristic search were automatically obtained applying the Neighbor-Join and BIONJ algorithms to a matrix of pairwise distances estimated using the Maximum Composite Likelihood (MCL) approach, and then selecting the topology with a superior log likelihood value. A discrete Gamma distribution was used to model evolutionary rate differences among the sites [5 categories (+*G*, parameter = 2.6875)]. The tree is drawn to scale, with branch lengths representing the number of substitutions per site. The analysis utilized 702 nucleotide sequences. All positions with less than 95% site coverage were eliminated. Fewer than 5% alignment gaps, missing data, and ambiguous bases were allowed at any position. Evolutionary analyses were conducted in MEGA7 [2]

**Fig. 4:**
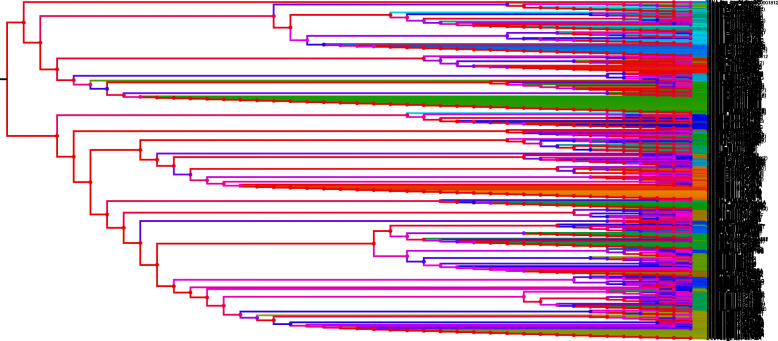
Recombination events in tRNA isotypes. Results indicated that tRNAs haves undergone dynamic recombination events during the course of evolution. The recombination study was conducted using IcyTree software using a nwk file format obtained from the phylogenetic tree

**Fig. 5:**
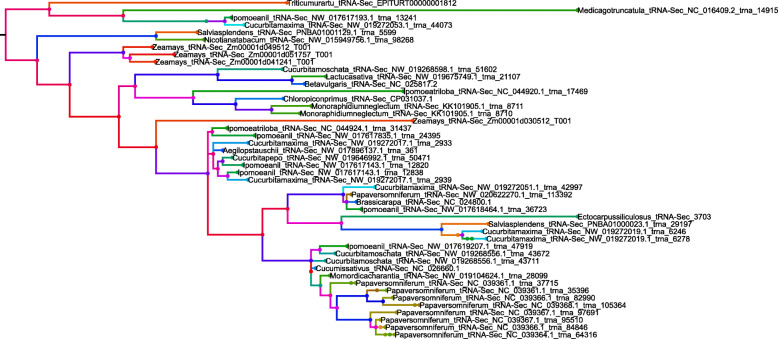
Recombination events in tRNA^Sec^ genes. Results indicate that tRNA^Sec^ have undergone recombination events during the evolution. The recombination study was conducted using IcyTree using the nwk file format of the phylogenetic tree of the tRNA^Sec^

A phylogenetic tree was constructed to investigate the evolution of *tRNA*^*Sec*^ genes by considering the nucleotide sequences of all the 20 tRNA genes along with *tRNA*^*Sec*^ genes. The phylogenetic tree revealed the 28 major tRNA groups (Fig. 3). The *tRNA*^*Sec*^ genes were clustered in the middle of the phylogenetic tree and *tRNA*^*Sec*^ was found to be present in at least six different clusters (Fig. 3). A few *tRNA*^*Sec*^ genes were grouped with *tRNA*^*Lys*^ (CUU), *tRNA*^*Asn*^ (GUU), *tRNA*^*Arg*^ (UCG, CCG), *tRNA*^*Gly*^ (UCC), and *tRNA*^*Trp*^ (CCA) (Fig. 3). The analysis indicates that tRNA^Sec^ is distributed in different clusters in the phylogenetic tree. This explains the role of duplication events in the evolution of *tRNA*^*Sec*^ genes. Therefore, an analysis was conducted to investigate the deletion/duplication events related to *tRNA*^*Sec*^ genes. As a result, we found that tRNA^Sec^ deletion events occurred more frequently than duplication events. A total of 45 duplications, 119 deletions, and 9 co-divergent events were identified within 68 *tRNA*^*Sec*^ genes found in 22 species (Supplementary Figure 2). The role of recombination in the evolution of *tRNA*^*Sec*^ was further analysed. Results indicated that *tRNA*^*Sec*^ genes had undergone recombination events, as did other *tRNA* genes (Fig. 4). The role of recombination and duplication of *tRNA*^*Sec*^ genes resulted in the sharing of its genetic sequence with other tRNAs genes which may perhaps explain why *tRNA*^*Sec*^ was present in different clusters within the phylogenetic tree. A recombination analysis of *tRNA*^*Sec*^ genes indicated the role of recombination events within the *tRNA*^*Sec*^ itself (Fig. 5). A time tree analysis revealed that the divergence time of *tRNA*^*Sec*^ genes in plant species occurred at least 2466.30 million years ago (MYA) (Supplementary Figure 3) and less than a MYA in the case of the *tRNA*^*Sec*^ in *P. somniferum*. The tRNA^Sec^ in *P. somniferum* was found to arise from a duplication event. The recent divergence time for the tRNA^Sec^ in *P. somniferum* indicates that this gene has undergone a recent duplication event.

### tRNA^Sec^ underwent a switch in anti-codons during evolution

tRNA genes undergo rapid changes during the course of their evolution to meet translational demand. Therefore, an attempt was made to better understand the role of *tRNA*^*Sec*^ genes in plant evolution. It is well known that the *tRNA*^*Sec*^ gene is encoded by a UCA anti-codon and that this gene was found in different clusters in the phylogenetic tree of tRNAs. An anti-codon switch occurs more frequently with a nucleotide sequence of a tRNA gene with a different anti-codon than with a gene with a similar anti-codon [51]. Therefore, the possibility of anti-codon switch in *tRNA*^*Sec*^ gene was examined. tRNA^Sec^ grouped with tRNA^Lys^ (CUU), tRNA^Asn^ (GUU), tRNA^Arg^ (UCG, CCG), tRNA^Gly^ (UCC), and tRNA^Trp^ (CCA). The UCA anti-codon of tRNA^Sec^ was replaced by CUU in tRNA^Lys^ and in tRNA^Asn^ it was replaced by GUU where the 2nd and 3rd nucleotide of the anti-codons were constant. In tRNA^Arg^ and tRNA^Gly^, the UCA anti-codon of tRNA^Sec^ was replaced by UCG and UCC where the 1st nucleotide of the anti-codons remained constant and the 2nd and 3rd anti-codons were variable. For the CCG anti-codon of tRNA^Arg^ and the CCA anti-codon of tRNA^Trp^, the 1st nucleotide of U (CA) of tRNA^Sec^ was replaced with a C nucleotide and the 3rd nucleotide remained variable.

### Statistical analysis

**Fig. 6:**
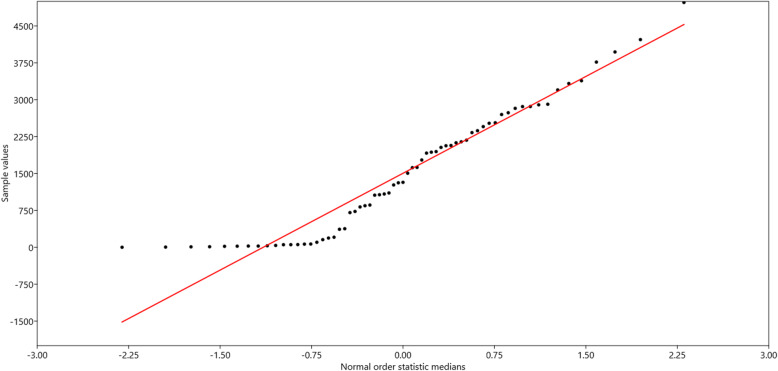
Normal probability plot of anti-codon numbers of the plant kingdom with correlation coefficient 0.9636 suggesting the datasets are normally distributed

The varied number and frequency of anti-codons led us to understand whether or not a dataset is approximately normally distributed. Therefore, we conducted normal probability plot study of anti-codon numbers (Fig. 6). The normal probability plot correlation coefficient was 0.9632. the correlation co-efficient and an approximately straight line indicate that normal distribution was good for the dataset (Fig. 6). Ordinary linear fit least square regression model of anti-codon numbers was conducted to find the best fit for a set of data by minimizing the sum of the offsets or residuals of points from the plotted curve and to understand the behaviour of dependent variables (Supplementary Figure 4). The method estimates the relationship by minimizing the sum of the squares in the difference between the observed and predicted values of dependent variable configured as a straight line. At 95% significance and intercept at zero, the slope was found to be 34.621 (Supplementary Figure 4). The statistical result of the ordinary least square regression was; *t* = 10.728, standard error *a* = 3.227, and *p* (slope) = 6.161E-16. For 95% bootstrap confidence interval (*N* = 1999); correlation *r* = 0.00916, *r*^*2*^ = 8.3917E-05, *t* = 0.072713, *p* (uncorr) = 0.94226, and pemutation *p* = 0.9404. the residual standard error of estimate was 147.

## Discussion

tRNA is an adaptor molecule that becomes charged when it binds an amino acid and subsequently donates it to an elongating peptide chain as determined by a codon-anti-codon recognition system. Each tRNA contain a characteristics anti-codon sequence which dictates the translation of a mRNA sequence into a protein. In some cases, the same codon can get decoded by different tRNA species and the same tRNA species can also become decoded by different codons due to wobble interactions (Watson-Crick base pairing) at the first position of an anti-codon and third position of the codon [26–28]. In our analysis of 128 species of the plants, none were found to encode all 64 anti-codons, which suggests that wobble base pairing exists in all plant species. The wobble interaction occurs at the G:U (guanine-uracil) base pairing and modifications in anti-codons that change the specificity of a codon [57–59]. Due to this redundancy, it is not necessary for a plant genome to encode all of existing anti-codons and utilize different tRNAs according to the requirement. The presence of only 29 anti-codons in the genome of *Klebsmordium nitens* and 31 anti-codons in *Bathycoccus prasinos*, however, are somewhat very interesting. Species *K. nitens* and *B. prasinos* belonged to the phylum algae and the genome sizes of these species are much smaller than the genome sizes found in gymnosperm and angiosperms. The absence of a greater number of anti-codons in these species suggests that the rate of wobble base-pairing might be quite high in these species. Mohanta et al., (2020) reported that species of cyanobacteria possessed 32 to 43 anti-codons per genome [20]. Cyanobacterial genomes are smaller than genomes of alae and higher plants [60]. The absence of a greater number of anti-codons in species with smaller genome is directly related to a higher frequency of wobble base-pairing. *Ipomea nil* (59), *Ipomea triloba* (58), *Papaver somniferum* (59), *Cucurbita pepo* (56), and *Zea mays* (59) possess a high number of anti-codons and so the occurrence of wobble base pairing may be quite minimal in these species. It will be interesting to determine the factors responsible for the occurrence of high and low frequencies of wobble base-pairing. Zhang et al., [61] reported that the presence of high concentration of amino acids in the nutrient media led to higher rate of mismatch incorporation of amino acids into the translating protein chain [61]. They also reported that wobble codon position is less stringent in base pair mismatch and base change in 3rd position explained additional 25% misincorporation either by favourable G^mRNA^/U^tRNA^ mismatch or wobble position mismatch [61]. The G/U mismatch was predominant during the codon recognition and which is commonly found in the nucleic acid secondary structures as well [62–64].

The abundance of the CAU anti-codon that encodes tRNA^Met^ was the greatest among all of the anti-codons (Supplementary File 1). Methionine is used to initiate the start of a polypeptide chain, and as a result, almost all proteins require a methionine amino acid. Therefore, the abundance of an anti-codon for tRNA^Met^ was found to be the highest. Additionally, tRNA^Met^ (CAU) was found to have evolved earlier than other tRNAs during the course of evolution [18, 19]. If the abundance of isoacceptors is considered, tRNA^Leu^, which contain six isoacceptors (GGA, AGA, CGA, UGA, ACU, GCU), has the highest abundance (7.808% of the collective plant species). Similarly, tRNA^Ser^, and tRNA^Arg^, both with six isoacceptors, have a high percentage of anti-codon abundance. This finding led us to conclude that, the higher the number of isoacceptors for tRNA isotypes, the greater the level of anti-codon sharing in a genome. The study also reveals that plant genomes encode tRNA^Leu^, tRNA^Ser^, and tRNA^Arg^ more frequently than other tRNAs. A proteome-wide analysis by Mohanta et al., [19] reported a higher abundance of Leu amino acids in the proteomes of the Plant Kingdom [65]. This observation directly corroborates that the number and abundance of *tRNA*^*Leu*^ genes in genome is directly proportional to the number of Leu amino acids in the proteome. In contrast, a few anti-codons, including GCG, GAG, GGG, GGC, ACU, ACC, UCA (Sec) (group E) of different tRNA isotypes were found to have a low abundance (Fig. 2). Yona et al., [51] reported that multiple copies of rare tRNAs are deleterious to a cell [51]. They also stated that the effective gene copy number of each tRNA anti-codon set can undergo changes during evolution that may be due to the changes in demand-to-supply [51]. A single point mutation in an anti-codon can change one tRNA to another. The lowest encoding anti-codon GCG of tRNA^Arg^ may have undergone a point mutation resulting in tRNA^Arg^ with ACG, CCG, and UCG, which avoids the deleterious effect of the GCG anti-codon. Previous studies have also noted that rare tRNAs may be essential for co-translational folding as low abundance could provide a pause in translation [44, 66].

When plants grow in a multitude of environmental conditions, environmental stress can induce the expression of genes needed for stress adaptation, which may affect codon usage by the transcriptome. This leads to a demand for a different pool of tRNAs to support the change in codon usage and avoid a translational imbalance [52, 67]. If the altered environmental conditions persist, the tRNAs have to undergo changes in their level of expression to meet and respond to the environmental stress-induced changes in gene expression. If the changes in supply-demand continue, it may lead to changes in the genetic pool of the tRNAs that are beneficial and favoured by selection pressures. These novel translational demands can be maintained by shifting nucleotides in the anti-codons rather than by the duplication of genes. The tRNA pool can evolve to maintain the translational requirement by adjusting the number and/or ratio of tRNA isotypes encoding the same amino acid. An anti-codon switch, however, can also dramatically change the ratios of tRNA isoacceptor within a tRNA pool. This can be done by increasing the copy number of one isoacceptor at the expense of others. The high sequence similarity of different anti-codons (anti-codon switch) can be the result of purifying selection that maintains sequence similarity. Sequence similarity, however, can result from concerted evolution that maintains sequence similarity through frequent recombination among members of the same gene family [68, 69]. The presence of a high level of recombination in tRNAs indicates that the evolution of plant tRNAs for anti-codon switch and sequence similarity may be due to concerted evolution. A single point mutation in an anti-codon can result in the encoding of a different tRNA family. It would be interesting to understand the evolutionary constraints that lead to the generation of more members while others have fewer members. It has been previously reported that tRNA^Leu^ encodes a higher number of tRNA genes in the genome, a feature that is directly related to the higher number of tRNA isoacceptors in tRNA^Leu^ [17–20]. The question remains if purifying selection plays a role in maintaining a low level of certain tRNAs, such as tRNA^Sec^, tRNA^His^, tRNA^Trp^, and tRNA^Tyr^. It is plausible that this purifying selection might be responsible for maintaining the anti-codons of these tRNAs at non-optimal levels. A previous study reported that increasing the copy number of a low copy tRNA gene family in a cell results in proteotoxic stress due to problems in protein folding [51]. In addressing the need for environmental adaptation, tRNA isotypes provide evolutionary plasticity to changes in translational demand due to their presence as a multi-member gene family. A few species have lost tRNA genes for particular tRNA isotypes and anti-codon switch/point mutations of anti-codons may be a factor that contributes to maintaining the function of a genome in the complete absence of a particular gene family.

Selenocysteine (a selenium containing cysteine analog) is co-translationally inserted in a small fraction of proteins (selenoproteins) and is driven by a tRNASec gene. Although Sec is found in all three domains of life, it is not universal. Approximately 20% of the prokaryotic genome contains selenoproteins, while in eukaryotes selenoproteins are reported to be more concentrated in the metazoan lineage [70–73]. The absence of selenoproteins in fungi and land plants has also been reported previously [74]. and results from a lack of a tRNA^Sec^ gene in their genomes. tRNA^Sec^ is encoded by a UGA anti-codon which also encodes a stop codon. A highly sensitive and efficient method of tRNA identification is needed to find tRNA^Sec^. The lack of suitable identification techniques may be the main reason for stating the absence of tRNA^Sec^ genes in fungal and plant genomes. Using current technology, however, we were able to identify tRNA^Sec^, as well as tRNASec genes in a few of the genomes of the analysed plant species.

## Conclusion

The repertoire of tRNA has a significant impact on the fitness of an organism. The frequency (abundance) of anti-codons that explains synonymous codon usage in coding genes, however, has remained unexplored. Anti-codon frequency can be directly attributed to the frequency of synonymous codon usage and an anti-codon table of the Plant Kingdom, along with the percent abundance of each anti-codon, can be very helpful for understanding the relationship between codon and anti-codon frequency in the genome. The 21st amino acid, selenocysteine, encoded by tRNA^Sec^ has undergone a duplication event along with an anti-codon switch. Understanding the mechanisms involved in the loss of tRNA genes in a few species may be crucial to deciphering the translation mechanism in these species. The frequency of the anti-codons GCG (Arg), GAG (Leu), ACU (Ser), GGG (Pro) were very low in abundance and appear to be the rarest form of anti-codons in the Plant Kingdom. Yona et al., [51] reported that multiple copies of rare tRNAs are deleterious to a cell [51], which suggests that large copy numbers of CGC, GAG, ACU, and GGG anti-codons may be deleterious to plant cells. Therefore, a very low number of these anti-codons are encoded in the plant genome. A few species have completely lost specific tRNA isotype genes in their genome. Additionally, a previous also reported the loss of tRNA genes in some plant genomes [75].

## Supplementary Information

**Additional file 1:** **Supplementary File 1.** Percentage frequency of anti-codons of the plant kingdom.

**Additional file 2:** **Supplementary Figure 1.** Multiple sequence alignment of plant tRNA^Sec^ genes. Alignment revealed the presence of conserved nucleotide sequences in the anti-codon loop and pseudo-uridine loop region (marked red). The Multiple sequence alignment was conducted using Multalin software (http://multalin.toulouse.inra.fr/multalin/).

**Additional file 3:** **Supplementary Figure 2.** Deletion, duplication, and codivergence events in tRNA^Sec^ in 128 analysed plant species. The gene tree of tRNA^Sec^ was reconciled with the species tree to identify deletion, duplication, and codivergence events in tRNA^Sec^ genes. Results of the analysis indicated that deletion events in tRNA^Sec^ were predominant over duplication and co-divergence events. Analysis was conducted using Notung software version 2.9.

**Additional file 4:** **Supplementary Figure 3.** Evolutionary time tree of tRNA^Sec^ genes. The analysis revealed that tRNA genes in the Plant Kingdom arose at least 2466.30 million years ago. The reference time period was considered based on the evolutionary time scale of the species *Chloropicon primus* and *Ectocarpus siliculosus* as per the time tree database (http://www.timetree.org/). The time tree shown was generated using the RelTime method. Divergence times for all of the branching points in the topology were calculated using the Maximum Likelihood method based on the Kimura 2-parameter model. Bars around each node represent 95% confidence intervals which were computed using the method described in Tamura et al. (2013) [76]. The estimated log likelihood value of the topology shown is − 1964.5432. A discrete Gamma distribution was used to model evolutionary rate differences among the sites [5 categories (+G, parameter = 2.8271)]. The tree is drawn to scale, with branch lengths representing the relative number of substitutions per site. The analysis utilized 68 nucleotide sequences. All positions with less than 95% site coverage were eliminated. Fewer than 5% alignment gaps, missing data, and ambiguous bases were allowed at any position. Evolutionary analyses were conducted in MEGA7 [56].

**Additional file 5:** **Supplementary Figure 4.** Ordinary least square regression between anti-codons and their numbers in the plant kingdom. The ordinary least square regression parameters (slope and intercept) and statistical significance of each regression are indicated. The solid red line represents linear least square fit and blue lines represented 95% confidence interval.

## Data Availability

All the studied data were taken from publicly available databases and data associated with the manuscript is provided in supplementary file.

## Abbreviations

tRNA: Transfer RNA
Ala: Alanine
Arg: Arginine
Asn: Asparagine
Asp: Aspartic acid
Cys: Cysteine
Gln: Glutamine
Glu: Glutamic acid
Gly: Glycine
His: Histidine
Ile: Isoleucine
Leu: Leucine
Lys: Lysine
Met: Methionine
Phe: Phenylalanine
Pro: Proline
Ser: Serine
Thr: Threonine
Trp: Tryptophan
Tyr: Tyrosine
Val: Valine
Sec: Selenocysteine
Pyl: Pyrrolysine
NCBI: National Center For Biotechnology Information
UPGMA: Unweighted pair group with arithmetic mean
*r*: Correlation coefficient
