# Supplementary material for: Construction of anti-codon table of the plant kingdom and evolution of tRNA selenocysteine (tRNASec)
Source: BMC Genomics. 2020 Nov 19;21:804. doi: 10.1186/s12864-020-07216-3 (PMC7678280; doi:10.1186/s12864-020-07216-3)
Supplement: Supplementary file 2 — Additional file 2: Supplementary Figure 1. Multiple sequence alignment of plant tRNASec genes. Alignment revealed the presence of conserved nucleotide sequences in the anti-codon loop and pseudo-uridine loop region (marked red). The Multiple sequence alignment was conducted using Multalin software (http://multalin.toulouse.inra.fr/multalin/). [file 12864_2020_7216_MOESM2_ESM.pdf]

```
NC_039358   GAAUCUGUGG CACAAUGG-- UAGUGC-GUC UGACAUCAAG UUAGAAGGUU GUG------- -----UGUUC AAUUC-ACAC CAGAUUCA
NC_039358   GAAUCUGUGG CACAAUGG-- UAGUGC-GUC UGACAUCAAG UUAGAAGGUU GUG------- -----UGUUC AAUUC-ACAC CAGAUUCA
NC_039366   GAAUCUGUGG CACAAUGG-- UAGUGC-GUC UGACAUCAAG UUAGAAGGUU GUG------- -----UGUUC AAUUC-ACAC CAGAUUCA
NC_039362   GAAUCUGUGG CACAAUGG-- UAGUGC-GUC UGACAUCAAG UUAGAAGGUU GUG------- -----UGUUC GAUUC-ACAC CAGAUUCA
NC_039364   GAAUCUGUAG CACAAUGG-- UAGUGC-GUC UGACAUCAAG UUAGAAGGUU GUG------- -----UGUUC AAUUC-ACAC CAGAUUCA
NC_039366   GAGUCUGUGG CACAAUGG-- UAGUGC-GUC UGACAUCAAA UCAGAAGGUU GUC------- -----UGUUC GAUUC-ACAC CAGAUUCA
NW_0206343  GAGUCUGUGG CACAAUGG-- UAGUGC-GUC UGACAUCAAA UCAGAAGGUU GUG------- -----UGUUC GAUUC-ACAC CAGAUUCA
NC_039367   GAGUCUGUGG CACAAUGG-- UAGUGC-GUC UGACAUCAAG UCAGAAAGUU GUG------- -----UGUUC GAUUC-ACAC CAGAUUCA
NC_039367   GUGUCUGUUG CACAAUGG-- UAGUGU-GUC UGACUUCAAG UCAGAAGGUU GUG------- -----UGUUC GAAAC-ACAC CAGAUUCA
NC_039360   GGAACUGUGG CGCAAUGG-- UGGCGC-AUC UGACUUCAAG UCAGAAGGUU ACG------- -----UGUUC GAUUC-ACGU CAGGUCCA
NC_039361   GGAACUGUGG CGCAAUGG-- UGGCGC-AUC UGACUUCAAG UCAGAAGAUU ACG------- -----UGUUC GAUUC-ACGU CAGGUCCA
NC_026660.  GGAUCCGUGC CGCAAUGG-- UAGCGC-GUC UGACUUCAGA UCAGAAGGUU GCG------- -----UGUUC GAUUC-ACGU CGGGUUCA
NW_0176174  GGAUCCGUGG CGCAAUGG-- UAGCGC-GUC UGACUUCAGA UCAUAAGGUU GCG------- -----UGUUC GAUUC-ACGU CGGGUUCA
NW_0192685  GGAUCUGUGG CGCAAUGG-- UAGCGC-GUC UGACUUCAGA UCAGAAGGUU GCG------- -----UGUUC GAUUC-ACGU CAGGUUCA
NW_0191046  GGAUCUGUGG CACAAUGG-- UAGCGC-GUC UGACUUCAGA UCAGAAGGUU GCG------- -----UGUUC GAUUC-ACGU UAGGUUCA
NW_0192685  GAAUUUGUGG CGUAAUGG-- UAGCGC-GUC UGACUUCAGA UCAGAAGGUU GUG------- -----UGUUC GAUUC-ACGU CGGGUUCA
NW_0176192  GAAUUCGUGG CGCAAUGG-- UAGCGC-GUC UGACUUCAGA UUAGAAGGUU GCG------- -----UGUUC AAUUC-ACGU UUGGUUCA
NC_039361   GAAUCUGUGG UGCAAUGG-- UAGCAC-GUC UGACUUCAAC GCAAAAAAU- GCG------- -----UGUUC GAUUC-ACGC CAGGUUCA
NW_0206196  GAAUCUGUGG UGCAAUGG-- UAGCAC-GUC UGACUUCAAG GCAGAAAAU- GCG------- -----UGUUC GAUUC-ACGC CAGGUUCA
NW_0206223  GAAUCUGUGG UGCAAUGG-- UAGCAC-GUC UGACUUCAAG GCAGAAAAU- GCG------- -----UGUUC GAUUC-ACGC CAGGUUCA
NW_0206227  GAAUCUGUGG UGCAAUGG-- UAGCAC-GUC UGACUUCAAG GCAGAAAAU- GCG------- -----UGUUC GAUUC-ACGC CAGGUUCA
NC_039366   GAACCUGUGG UGCAAUGG-- UAGCAC-AUA UGACUUCAAG UCAGAAGAU- GCG------- -----UGUUC GAAUC-ACGC CUGGUUCA
NW_0206188  GAACCUGUGG UGCAAUGG-- UAGCAC-AUA UGACUUCAAG UCAGAAGAU- GCG------- -----UGUUC GAAUC-ACGC CUGGUUCA
NC_039367   GAACUAGUGG UGCAAUGG-- UAGCAC-GUC CGACUUCACG UCGGAAGAU- GCG------- -----UGUUC GAAUC-ACGU CUGGUUCA
NC_039368   GAACUAGUGG UGCAAUGG-- UAGCAC-GUC CGACUUCACG UCGGAAGAU- GCG------- -----UGUUC GAAUC-ACGU CUAGUUCA
NW_0206222  GGGUCCAUAG CUCAGUGG-- UAGAGC-AUU UGACCUCAGA UCAAGAGGUC ACC------- -----GGUUC GAACC-CGGU UGGGCCCU
NC_024800   GGGUCCAUAG CUCAGUGG-- UAGAGC-AAU UGACUUCAGA UCAAUAGGUC ACC------- -----GGUUC GAACC-CGGU UGGGCCCU
NW_0176184  GGGUCCAUUG CUCAGUGG-- UAGAGC-AUU UGACUUCAGA UCAAGAGGUC ACC------- -----AGUUC GAACC-UGGU UGGGCCCU
NW_0192720  GGGCCUAUAG CUCAGUGG-- UAGAGC-AUU UGACUUCAGA UCAAAAGGUC ACC------- -----GCUUC AAACC-CGAU UGGGCUCU
NW_0192720  GACCACAUAG CGUAGUGGAU UAGCAC-AUC UGACUUCAGA UUAGAAGGUC AUG------- -----GGUUU GACUC-CCAU UGUGGUUG
NW_0196469  GACCGCAUAG CGUAGUGGAU UAGCGU-GUC UGACUUCAGA UCAGAAGGUC GUG------- -----GGUUC GACUC-CCAC UGUGGUCG
NW_0176171  GACCGUAUAG CGCAGUGGAU UAGAGU-GUC UGACUUCAGA UCAAAAGGUC GUG------- -----GGUUC GACUC-CCAC UGUGGUUG
NC_044924   GACCACAUAG UGCAGUGGAU UAGCGC-GUU UGACUUCAGA UCAAAAGGCC GUG------- -----GGUUC GACUC-CCAU UGUGGUCA
NW_0176178  GGUUGCAUAG UGCAGUGGAU UAGUGC-GUC UGACUUCAAA UUAGAAGAUU AUG------- -----GGUUC GACUC-CCAU UGUGGUCA
NW_0114998  GCGUUUGUAG UCCAACGG-U UAGGAU-AAU UGCCUUCAAA GCAAUAGACC -CG------- -----GGUUC GACUC-CCGG CAGACGCA
NW_0176181  GCGUUUGUAG UCCAACGG-U UAGGAU-AAU UGCCUUCAAA GCAAUAGACC -CG------- -----GGUUU GACUC-CCGG CAAACGCA
NW_0192720  GCGUUUGUAG UCCAACGG-U UAGGAU-AAU UGCCUUCAAA GCAAUAGACC -CG------- -----AGUUC AACUC-CCGG CAAAUGAA
PNBA010000  GUGUUUGUAG UCCAAUGG-U UAUGAU-UAU UGCCUUCAAA GCAAUAGACC -UG------- -----GGUUC GACCCUCCGG GCACC
PNBA010011  AUAUUCUUAG UUCAGUUCGA UAAAAAUGUG GGUCUUCAAA ACUCGAUGUC GUA------- -----GGUUC AAAUC-CUAC AUAAACA
NW_0159497  GCACUCUUAG UUCAGUUCGG UAGAAC-GUG GGUCUUCAAA ACCCAAUGUC AUA------- -----GGUUC AAAUC-CUAC AGAGCGUG
NC_044920   GGGGUGGUGG CGCAGUUGGC CAGCGC-GCG GGUAUUCAGC ACCUGAGGUC GAG------- -----AGUUC GAAUC-UCUC CCACCCCA
NW_0192720  GUCUGGGUGG UGUAGUUGGU UAUCAC-GCU AGUCU--CAC ACUAGAGGUC CUC------- -----AGUUU GAACC-UUGG CUCAGACA
NC_036649   GUCUGGGUGG UGUAGUUGGU UAUCAC-GCU AGUCU--CAC ACUAGAAGUC CCC------- -----GGUUC AAACC-CAGG CUCAGACA
NW_0176171  GUCUGGGUGG UGUAGUUGGU UAUCAC-GCU AGUCU--CAC ACAAGAGGUC CCC------- -----AGUUC AAACC-UGGG CUCAGACA
NC_025817   GGCGGCAUGG CC--GAGUGG U-AAGGCGGA GGACUUCAAA UCCUUGUUCC CU-------- -----AGUUC AAAUCCGGGU GUUGCUU
NW_0196757  GGUGGCAUGG CC--GAGUGG U-AAGGCGGG GGACUUCAAA UCCUUUUUCC CU-------- -----GGUUG AAAUCCGGGU GCCACCU
CP031037.1  GCGGGACUGA CCCCUGGCGG -CCAGGGCGC GGACUUCAAA UCCGUGGCGG CCGCGAGGCC GAGC-GGUUC GACUCCGCCU GA
KK101905.1  GCCAGGAUGA ACCCAGGUGG UCCUGGGCGC GGACUUCAAA UCCGUGGCGG CCGAGAGGCC GAGU-GGUUC AAUUCCACCU UCCCGGCG
KK101905.1  GCCACCAUGA UCCCAGCUGG UGCUGGGCGC GGACUUCAAA UCCGUGGCGG CCGAGAGGCC GAGU-GGUUC GAUUCCACCU UGGAGGCG
NW_0192685  GUUGAUAUGU UC--GAGUGG UUAAGGAGAA AGACUUCAAA UCUGUUG-GG CUUUGC--CC GCGUAGGUUC GAACCCUACU GUCGA
NC_016409     AGUUUUGA A---UAGUGG UUAACCUUCG GGUUUUCAAG UAAAAAGGAU CCGGUC--AA AAGAUAAAUA AAAUCCGACU AAGAAUC
```
