# Supplementary figures and images for: Construction of anti-codon table of the plant kingdom and evolution of tRNA selenocysteine (tRNASec)
Source: BMC Genomics. 2020 Nov 19;21:804. doi: 10.1186/s12864-020-07216-3 (PMC7678280; doi:10.1186/s12864-020-07216-3)

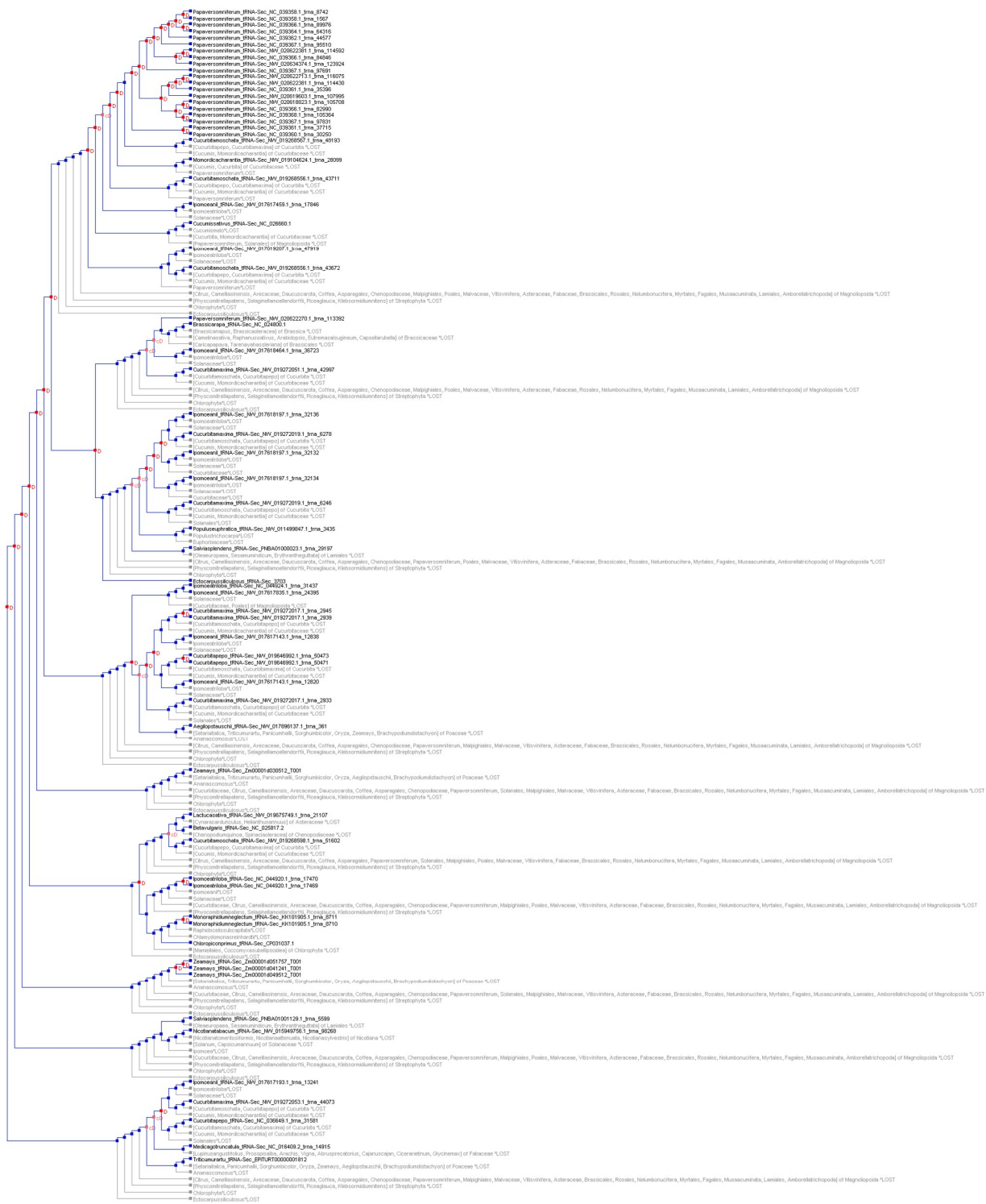

Supplement: Supplementary file 3 — Additional file 3: Supplementary Figure 2. Deletion, duplication, and codivergence events in tRNASec in 128 analysed plant species. The gene tree of tRNASec was reconciled with the species tree to identify deletion, duplication, and codivergence events in tRNASec genes. Results of the analysis indicated that deletion events in tRNASec were predominant over duplication and co-divergence events. Analysis was conducted using Notung software version 2.9. [file 12864_2020_7216_MOESM3_ESM.pdf]

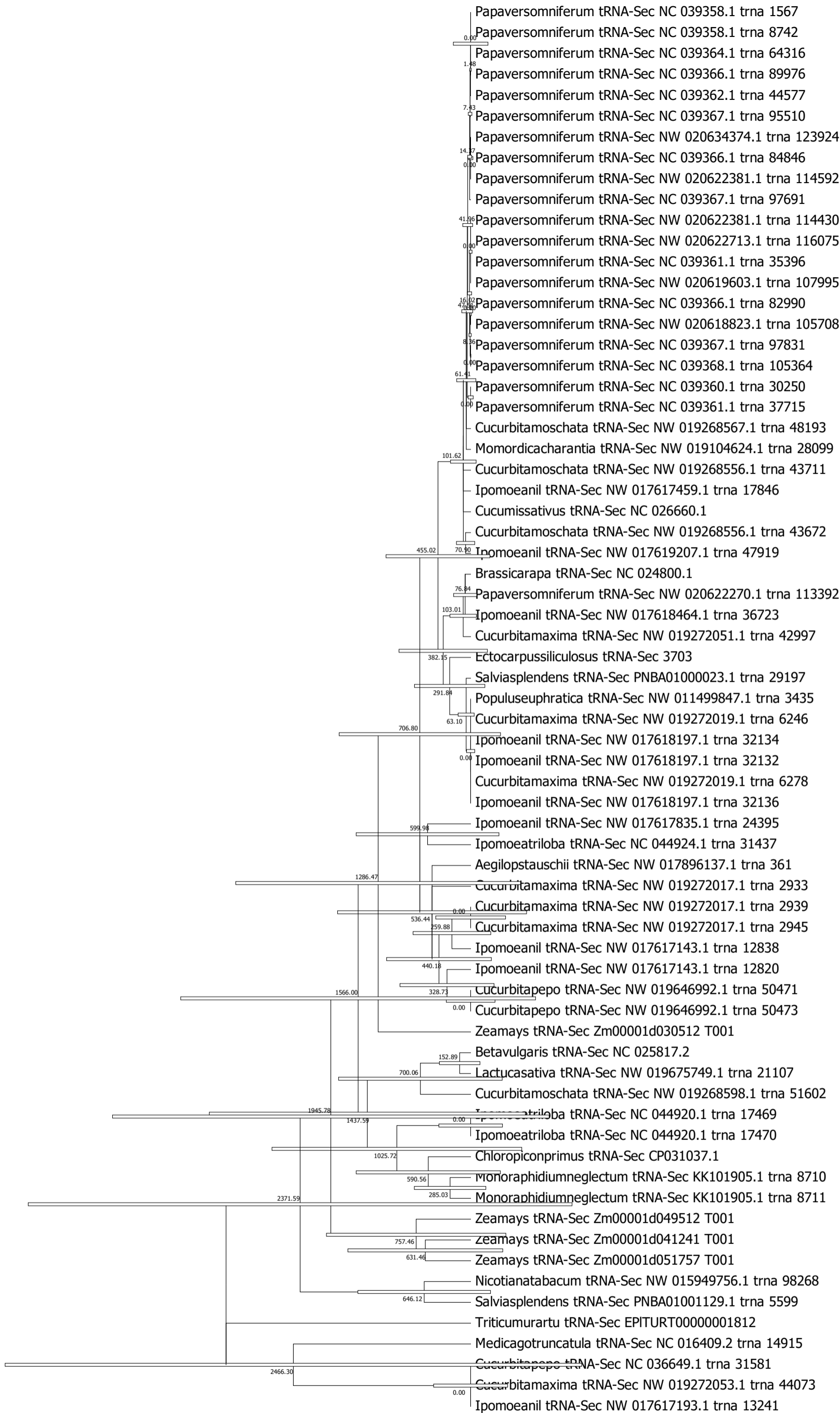

3000 2500 2000 1500 1000 500 0 Divergence Time

Supplement: Supplementary file 4 — Additional file 4: Supplementary Figure 3. Evolutionary time tree of tRNASec genes. The analysis revealed that tRNA genes in the Plant Kingdom arose at least 2466.30 million years ago. The reference time period was considered based on the evolutionary time scale of the species Chloropicon primus and Ectocarpus siliculosus as per the time tree database (http://www.timetree.org/). The time tree shown was generated using the RelTime method. Divergence times for all of the branching points in the topology were calculated using the Maximum Likelihood method based on the Kimura 2-parameter model. Bars around each node represent 95% confidence intervals which were computed using the method described in Tamura et al. (2013) [76]. The estimated log likelihood value of the topology shown is − 1964.5432. A discrete Gamma distribution was used to model evolutionary rate differences among the sites [5 categories (+G, parameter = 2.8271)]. The tree is drawn to scale, with branch lengths representing the relative number of substitutions per site. The analysis utilized 68 nucleotide sequences. All positions with less than 95% site coverage were eliminated. Fewer than 5% alignment gaps, missing data, and ambiguous bases were allowed at any position. Evolutionary analyses were conducted in MEGA7 [56]. [file 12864_2020_7216_MOESM4_ESM.pdf]

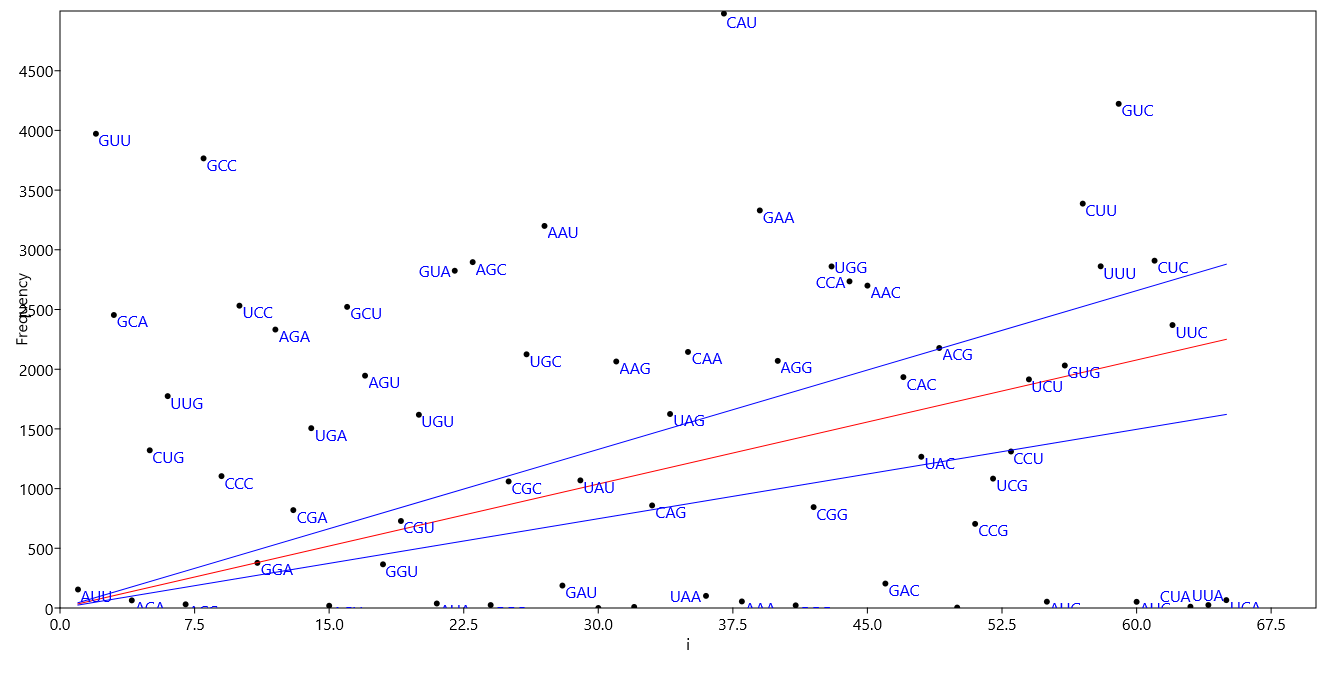

Supplement: Supplementary file 5 — Additional file 5: Supplementary Figure 4. Ordinary least square regression between anti-codons and their numbers in the plant kingdom. The ordinary least square regression parameters (slope and intercept) and statistical significance of each regression are indicated. The solid red line represents linear least square fit and blue lines represented 95% confidence interval. [file 12864_2020_7216_MOESM5_ESM.tif]
